# Genome Assembly and Population Sequencing Reveal Three Populations and Signatures of Insecticide Resistance of *Tuta absoluta* in Latin America

**DOI:** 10.1093/gbe/evad060

**Published:** 2023-04-18

**Authors:** Kyle M Lewald, Christine A Tabuloc, Kristine E Godfrey, Judit Arnó, Clérison R Perini, Jerson C Guedes, Joanna C Chiu

**Affiliations:** Department of Entomology and Nematology, University of California, Davis; Department of Entomology and Nematology, University of California, Davis; Contained Research Facility, University of California, Davis; IRTA, Cabrils, Spain; Department of Phytosanitary Defense, Federal University of Santa Maria, Brazil; Department of Phytosanitary Defense, Federal University of Santa Maria, Brazil; Department of Entomology and Nematology, University of California, Davis

**Keywords:** invasion biology, population history, insecticide resistance, *Tuta absoluta*, tomato, *Phthorimaea absoluta*

## Abstract

*Tuta absoluta* is one of the largest threats to tomato agriculture worldwide. Native to South America, it has rapidly spread throughout Europe, Africa, and Asia over the past two decades. To understand how *T. absoluta* has been so successful and to improve containment strategies, high-quality genomic resources and an understanding of population history are critical. Here, we describe a highly contiguous annotated genome assembly, as well as a genome-wide population analysis of samples collected across Latin America. The new genome assembly has an L50 of 17 with only 132 contigs. Based on hundreds of thousands of single nucleotide polymorphisms, we detect three major population clusters in Latin America with some evidence of admixture along the Andes Mountain range. Based on coalescent simulations, we find these clusters diverged from each other tens of thousands of generations ago prior to domestication of tomatoes. We further identify several genomic loci with patterns consistent with positive selection and that are related to insecticide resistance, immunity, and metabolism. This data will further future research toward genetic control strategies and inform future containment policies.

**Significance**

This study leveraged long-read sequencing to generate a highly contiguous genome assembly for the serious agricultural pest *Tuta absoluta*. Furthermore, population sequencing was conducted to test the long-standing hypothesis that *T. absoluta* spread across Latin America in the 20th century due to human transportation. Our results revealed that *T. absoluta* populations diverged before tomato agriculture, suggesting human transportation may not have been the major factor in the rapid spread of this pest across Latin America.

## Introduction

*Tuta absoluta* (also known as *Phthorimaea absoluta* [Chang and Metz 2021]) is a worldwide economic pest of tomatoes and other solanaceous crops. A member of the gelechiid family, this moth lays eggs on the aboveground portion of the plant, where the hatched larvae will spend their lives creating “mines” throughout the plant tissue before pupating and emerging as adults (Godfrey et al. 2018). At a reproduction rate of up to ten generations per year, untreated infestations will eventually result in complete death of the plant, leading to up to 100% agricultural loss. Although a large effort has been made to develop and implement integrated pest management (IPM) programs across different world regions (Desneux et al. 2022), typical treatments have included heavy use of a variety of insecticides (Siqueira et al. 2000), leading to the rapid appearance of insecticide resistance. As tomatoes represent a massive economic industry, with an estimated 252 million metric tons of tomatoes harvested in 2020 (FAOSTAT 2020), there is a serious need to understand the invasive biology of this insect and to develop tools for detection and prevention.

*Tuta absoluta* was originally detected in Peru in 1917 (Meyrick 1917) but was not recorded as an agricultural pest until the 1960s and 1970 s when it was discovered in tomato fields in Chile, Argentina, and Venezuela; by the 1990s, it was widespread across South America. In 2006, *T. absoluta* appeared in Spain (EPPO 2008); since then, it has rapidly colonized Europe, Asia, and Africa. It is generally believed that the Peruvian highlands is the ancestral home of *T. absoluta* and that the rapid colonization to the rest of Latin America was due to the introduction of *T. absoluta* by human transport of contaminated fruit, although few studies have confirmed this (Desneux et al. 2011). Previous research using mitochondrial and microsatellite DNA markers found some evidence of population structure, as well as evidence that the European invasion originated from a single population in central Chile (Cifuentes et al. 2011; Guillemaud et al. 2015). However, determination of higher-resolution population structure, migration events, divergence times, and population size can benefit from using a larger number of markers, such as what is produced from genome-wide sequencing studies (Trask et al. 2011; Willing et al. 2012; Rašić et al. 2014; Koch et al. 2020). Additionally, few genetic studies have been conducted to understand how *T. absoluta* has performed so successfully as an agricultural pest beyond targeted examinations of known insecticide resistance alleles. One reason for this has been the lack of a highly contiguous genome with annotated genes. A short-read–based assembly has been previously published for the purpose of developing molecular diagnostics (Tabuloc et al. 2019); however, it is highly fragmented and duplicated.

In this study, we addressed these issues by using long-read sequencing technology to produce a highly contiguous genome assembly for *T. absoluta*. We then use short-read technology to sequence genomes of individuals collected across Latin America, as well as a Spanish population, to identify single nucleotide polymorphisms (SNPs) in an unbiased manner. We use these SNPs to detect population structure and estimate population history parameters to understand how and when *T. absoluta* spread across Latin America. Finally, we use genome scanning statistics to identify genes putatively under selection that may explain *T. absoluta's* success as an agricultural pest. We expect that the genome assembly and population data will be an asset toward developing new strategies to manage this pest.

## Results

### New Pacbio *T. absoluta* Genome Assembly Improves Gene Annotation and Contiguity

Before performing any population analyses, we decided to produce a high-quality reference genome based on long-read technology. New protocols for PacBio HiFi sequencing allow for low DNA input, which was critical in our case as Lepidopterans are notoriously heterozygous and using DNA pooled from many individuals would make genome assembly challenging. We sequenced a single moth originating from a laboratory colony at the Institute of Agrifood Research and Technology (IRTA), Cabrils, Spain, and obtained 16.2 Gbp of sequence after collapsing circular consensus reads. Based on k-mer analysis with GenomeScope, the genome is 2.9% heterozygous and has 38.8% repeat content. GC versus k-mer plots show that there is likely no mass contamination from other species or microbes (supplementary fig. S1, Supplementary Material online). The k-mer-based haploid length estimate is 524 Mbp, which is close to the 564 Mbp estimate based on flow cytometry (Paladino et al. 2016).

**Fig. 1.**
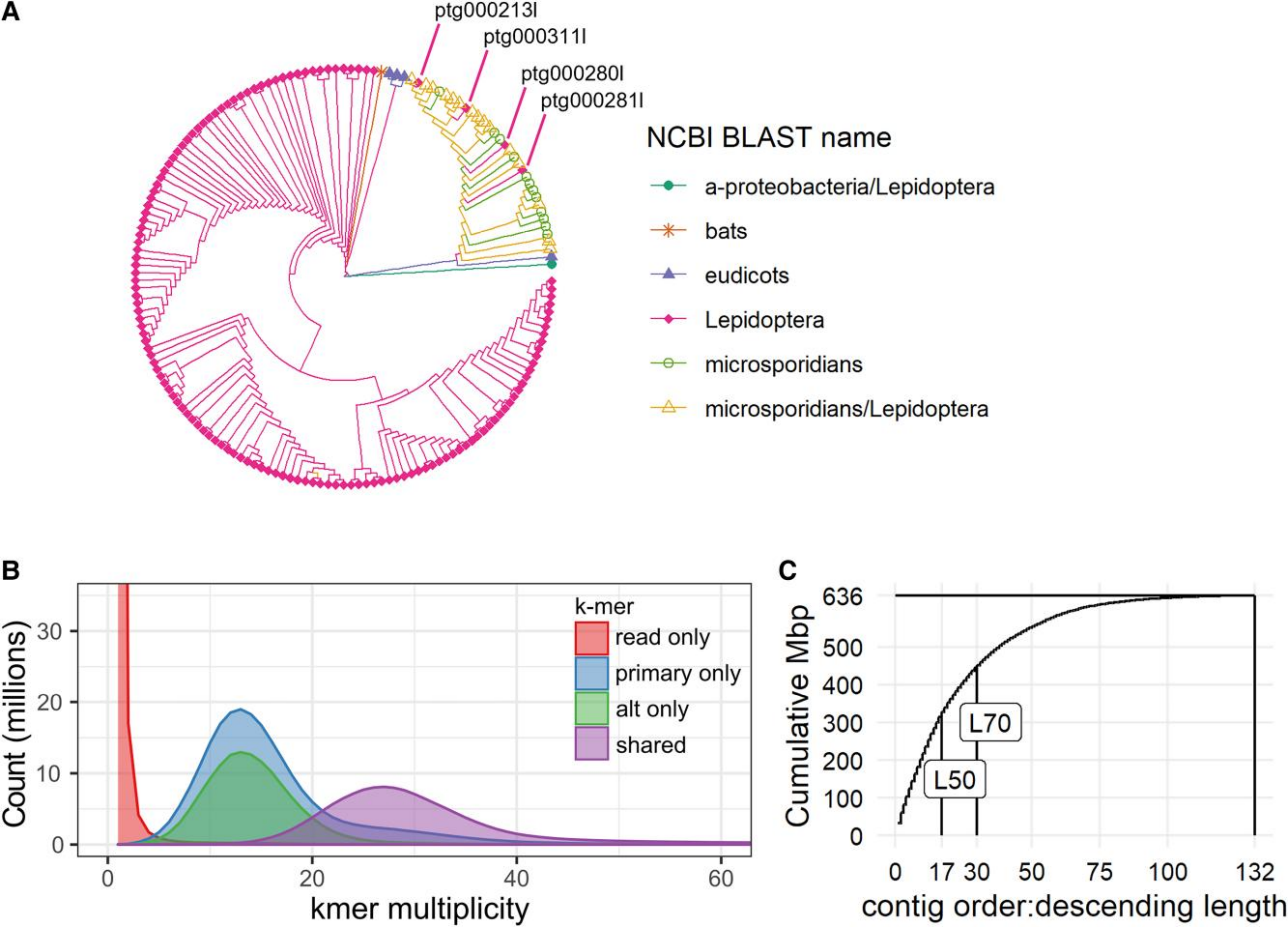
Genome assembly assessment. (*A*) K-mer-based distance tree of contigs in the primary genome assembly before decontamination, labeled by NCBI Blast matches in the “nt” database. Contig ptg000311*l* matched to insect mitochondrial sequences. Contigs ptg000311*l*, pg000280*l*, and ptg000281*l* were contigs annotated as “Lepidoptera” by BLAST but clustered with microsporidian sequences in the tree. (*B*) K-mer multiplicity plot of input CCS reads against the assembly. “Read only” indicates k-mers that only appear the raw reads; “primary only” “alt only” indicate read k-mers that appear in only one of the two haplotig sets; “shared” indicates k-mers that appear in both haplotig sets. (*C*) Cumulative length of contigs in primary assembly, ordered from the longest to the shortest. L50 and L70 indicate smallest number of contigs that contain 50% or 70% of the assembly length.

To assemble reads, we used the HiFi assembler hifiasm and compared quality and contiguity metrics using Merqury. The accurate long reads allow for the ability to separately assemble maternal and paternal haplotypes at heterozygous regions. While hifiasm attempts to separate assembled contigs into primary and alternate haplotypes, we found that the primary assembly still had high haplotype retention based on its 990-Mbp length and the large peak of raw read k-mers that appear twice in the assembly (supplementary fig. S2*A*, Supplementary Material online). We decided to further remove haplotigs using purge_dups, which shrunk the primary assembly size to 650.6 Mbp and eliminated the 2× raw read k-mer peak (supplementary fig. S2*B*, Supplementary Material online). Additionally, BUSCO analysis using the OrthoDB Lepidoptera gene set found the percent of complete, duplicated BUSCOs dropped from 48.5% in the unpurged assembly to 6.2% in the purged assembly (supplementary fig. S2*C*, Supplementary Material online). This means that improperly retained alternate haplotypes have been removed from the primary assembly. When we examine the raw read k-mer multiplicity in the primary assembly, we see a peak of k-mers that map only to the primary or alternate assembly at k = 13, which corresponds to the heterozygous portions of the genome (fig. 1*B*). We also see a peak at k = 26 in k-mers that are shared between the primary and alternate assemblies, which matches the expectation of a diploid genome with double the read coverage in any homozygous regions of the genome. The frequencies of k-mers missing from either assembly are low and represent k-mers from sequencing read errors.

To identify which contigs came from contaminant DNA, we used the Blast nt database, as well as a k-mer distance tree (fig. 1*A*) and found multiple contaminants. We identified a *Wolbachia* genome contig, several tomato contigs, and many microsporidian contigs, primarily from the *Nosema* genus (a common insect fungal parasite), all of which were expected. We also identified multiple contigs that matched to both *Nosema* and Lepidopteran queries in Blast. Based on the distance tree's clustering of these contigs with other *Nosema*-only contigs, as well as the GC content (supplementary fig. S3*A*, Supplementary Material online), we decided to exclude these from the assembly. We also noticed four contigs that matched only to Lepidopteran contigs but clustered with Nosema sequences in k-mer content. One of these, ptg000311, matched to Lepidopteran mitochondrial sequences and likely represents the *T. absoluta* mitogenome. The remaining three matched to the same *Papilio xuthus* genome assembly (PRJNA291600) but were likely the result of *Nosema* contamination in the assembly. In addition, these contigs’ GC and repeat content profiles were distinct from all other Lepidopteran contigs (supplementary fig. S3, Supplementary Material online), so we excluded them from the assembly. Finally, one contig matched to a mouse-eared bat (*Myotis*) mitochondrial genome; this was possibly contamination from the sequencing facility and was excluded as well.

After removing these contigs, our primary assembly contained 132 contigs all longer than 10 kb with a final length of 635.9 Mbp; 70% of the genome was captured in the 30 longest contigs (L70); as *T. absoluta* has 29 chromosomes, this suggests that our assembly is approaching chromosome-level contigs (fig. 1*C*). This represents a significant improvement from the previously published *T. absoluta* genome which consists of 81,653 contigs and a length of 906 Mbp (Tabuloc et al. 2019).

To generate gene models and functionally annotate genes, we used RepeatModeler and RepeatMasker to soft-mask the genome for repeats using both known Lepidopteran repeat sequences, as well as de novo sequences identified from our assembly. We followed with BRAKER2 to identify gene models and functionally annotated models with EnTAP, based on multiple protein databases (RefSeq Invertebrate, UniProt Swiss-Prot and TrEMBL, Lepbase, and EggNOG) and published *T. absoluta* RNAseq data sets. Of the 19,570 gene models identified by BRAKER2, 17,183 were identified as complete by gffread and EnTAP, with 14,019 transcript models matching to a gene name or functional annotation.

### Population Structure of *T. absoluta*

#### Population Sampling and Sequencing

**Fig. 2.**
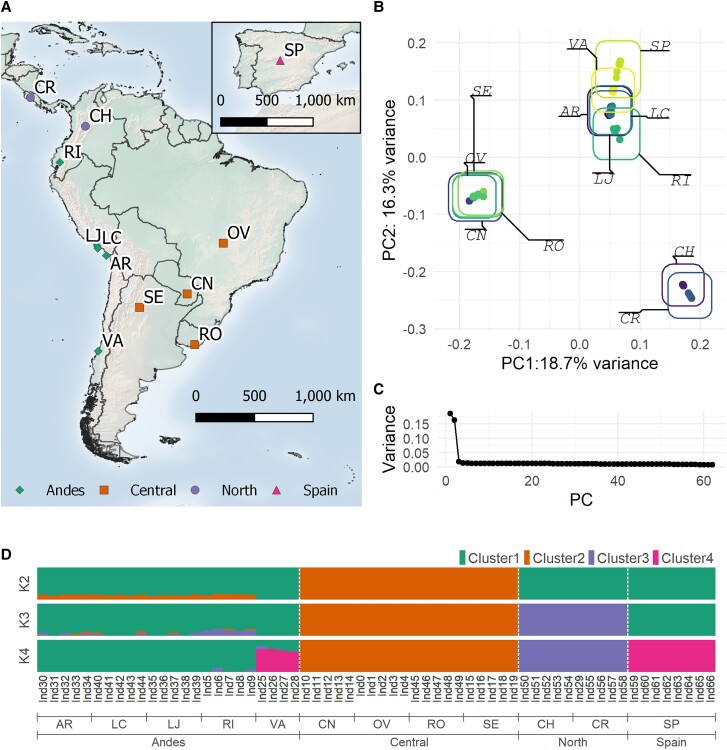
Sampling sites and population structure of *T. absoluta* individuals. (*A*) Map of sampling locations. Between four and eight individuals were sequenced per location. Legend indicates grouping as determined from PCA and admixture analyses. (*B*) PCA plot of the first two principal components (PCs), based on genotype likelihoods from 933,060 sites. (*C*) Percent variance captured by PCs in descending order. (*D*) Admixture analysis for two to four clusters. Colored bars indicate the posterior probability of an individual belonging to a given cluster. Location codes: AR, Arica, Chile; CH, Chia, Colombia; CN, Campo Novo, Paraguay; CR, Costa Rica; LC, La Curva, Peru; LJ, La Joia, Peru; OV, Ouro Verde, Brazil; RI, Riobamba, Ecuador; RO, Rocha, Uruguay; SE, Santiago del Estero, Argentina; SP, Barcelona, Spain; VA, Villa Alegre, Chile.

We analyzed whole-genome sequencing data from individuals collected in 2017 from field and greenhouse sites across South America and Costa Rica (CR), as well as a lab colony from Spain (Tabuloc et al. 2019) (fig. 2*A,*supplementary table S1). Mapping rates to the new genome assembly ranged between 70% and 90%, although sequencing depth per individual was low (between 1× and 17×) (supplementary fig. S4, Supplementary Material online). One population from Argentina (MP, Mar del Plata, Buenos Aires State) had extremely low mapping rates and read depth, so we excluded it from further analysis. Wherever possible, we used methods based on genotype likelihoods, rather than genotype calls, to account for uncertainty that results from the low read depth.

#### Three Distinct *T. absoluta* Populations Exist in Latin America

To investigate population structure in our samples, we used principal component analysis (PCA) based on allele frequencies from over 900,000 SNPs. The first two PCs captured 18.7% and 16.3% of the total variance in the data, with the remaining PCs each capturing <5% of the total data variance (fig. 2*C*). Samples primarily cluster together based on collection site but also formed three distinct regional groups (fig. 2*B*). Samples from Chile, Peru, and Ecuador form an “Andes” cluster west of the Andes Mountains; samples from Brazil, Uruguay, Paraguay, and Argentina form a “Central” cluster, east of the Andes Mountains; and samples from Colombia and CR form a “North” cluster. Spanish samples grouped tightly with the Andes cluster, particularly the Villa Alegre (VA, Chile) site.

When then performed admixture estimation to confirm this population clustering and detect evidence of admixture between regions. When three clusters were allowed in admixture estimation, samples group into the same three clusters as in PCA, while at four clusters, the Spanish samples become their own group, with VA samples sharing a large proportion of admixture. Compared with other Andes populations, the VA samples are more differentiated from Central and North sites as well, with little signal of admixture at all levels of k tested. The other Andes populations (AR, La Curva [LC], La Joia [LJ], and Riobamba [RI]) all had low admixture proportions from Central at k = 2, although at k = 3, we see that all RI, Ecuador, samples exhibited admixture from the North populations. This suggests that the non-VA Andes populations are more closely related to Central populations than VA and that VA could represent an admixture between the population that gave rise to the Spanish lineage and the other Andes populations. Additionally, we see that RI represents an intermediate population between the Andes and North, which makes sense given its geographic location between the two clusters.

To further quantify population structure between these clusters, we calculated nucleotide diversity, Tajima's *D*, and *F*st using genotype likelihoods (supplementary fig. S5, Supplementary Material online). For all clusters, nucleotide diversity was ∼2%, which is fairly high compared with most Lepidopterans (Mackintosh et al. 2019). If we look at the weighted *F*st, we see that the differentiation between clusters is high, particularly between North and all other clusters. The combination of high diversity levels and high *F*st could mean these regions diverged from each other a long time ago, prior to the detection of *T. absoluta* by growers across Latin America in the 1960s to 1980s. If divergence had occurred recently, we might expect reduced diversity levels in invasive populations relative to the ancestral population.

#### Treemix Confirms Clustering and Detects Migration Events to Ecuador and Chile

**Fig. 3.**
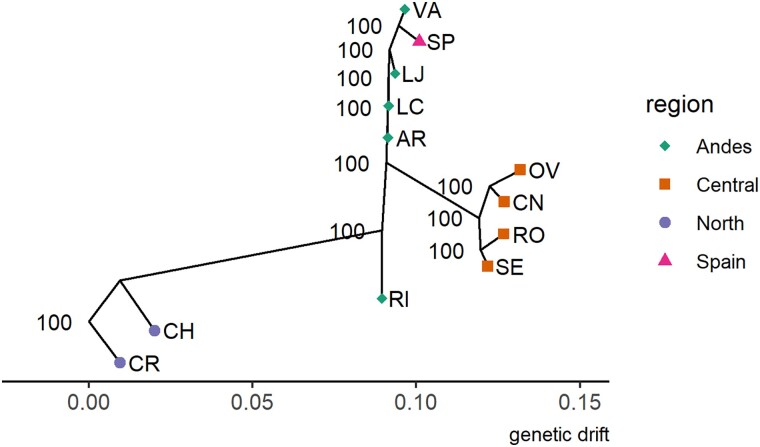
Maximum likelihood tree from Treemix with no migration edges called, rooted on the Costa Rica (CR) samples, based on 47,535 SNPs. Confidence values were based on 100 jackknife bootstraps with 500 SNP bins. *X*-axis represents relative genetic drift distance between populations.

To detect potential migration events between populations, we used Treemix to build a maximum likelihood (ML) tree based on allele frequencies and to predict migration edges. We also used Treemix to calculate *F*3 statistics for all population triplets. *F*3 statistics measure the level of genetic drift from a population “A” to the common node of populations “A,” “B,” and “C.” If the *F*3 statistic is negative, it indicates that population “A” is admixed from populations “B” and “C” (Reich et al. 2009). As Treemix was designed to take allele count data per population, we called genotypes using PCAngsd using a 95% accuracy cutoff and counted alleles within each sampling location. After filtering out loci with missing data, 47,535 SNPs were available for use. In general, the tree topography aligns with results from PCA and admixture analyses. We see sampled sites cluster into the same three clusters, North, Andes, and Central, with the Spanish samples sister to the VA (Chile) site (fig. 3). Within each region, branch lengths are short, indicating relatively little genetic drift between sampling sites. In agreement with *F*st estimates, North populations have experienced more genetic drift from the Andes and Central populations, compared with the Andes and Central populations with each other.

Interestingly, the RI (Ecuador) site does not form a clade with other Central populations but descends from the common ancestor of the Central/Andes group. Based on admixture analysis that showed low levels of admixture in RI from the North, the position of RI in the tree could be further evidence that Ecuador represents an intermediate mixing zone between populations North and South of it. To investigate further, we reran Treemix allowing between one and five migration events (*m* = 0–5) and calculated the *F*3 statistic between all combinations of three populations to see if admixture was supported. At *m* = 2, 4, and 5, Treemix reported a strong migration (between 11% and 25%) from the Spain or Spain/VA branch to RI, while at *m* = 3 and 5, Treemix reported a weak migration event (2–7%) from the North to RI (table 1). *F*3 statistics *F*_3_(RI; CH, SP) and *F*_3_(RI; CR, SP) were significantly negative (table 2), indicating that a simple bifurcating tree does not explain RI's relationship with CH, CR, and SP. Although Treemix infers a migration from the Spanish branch to the RI branch, it is important to remember that this migration is inferred to have occurred somewhere along the branch between the current day Spanish population and the most recent common ancestor of Spain and VA (Chile). This migration could have occurred early in the branch, when the population was still in Chile, or late in the branch, when the population moved to Spain. In 2006, Chile shipped over 29,000 kg of fresh tomatoes to Spain and imported no tomatoes from Spain, making it unlikely that *T. absoluta* was transported from Spain back to the RI population hitchhiking in tomato imports (Worldbank 2006). Thus, it is more likely that the source of the migration to RI was from the Chilean ancestor of the Spanish population.

**Table 1:** Migration Events with Predicted Weights from Treemix between Populations, under Different Models Allowing between 1 and 5 Migration Events (m)

m	Weight (%)	Start-Node	End-Node
1	8.8*	SP	AR
2	11.2*	SP	AR
2	15.1*	VA, SP	RI
3	17.7*	SP	AR
3	17.3*	SP	LC
3	2.0*	CR, CH	RI
4	10.6*	SP	AR
4	25.4*	VA, SP	RI
4	2.2*	OV	RI
4	13.6	SP	LJ
5	21.3*	SP	LC, AR
5	11.0*	SP	RI
5	24.2*	SP	LJ
5	6.7*	CR	RI
5	2.9*	LC, AR	OV

* indicates the migration significantly improved model fit (*P* < 0.05) based on the Wald statistic using jackknife estimates. Inferred migrations originate somewhere between the “start-node” and the start-node's most previous branchpoint and terminate somewhere between the end-node and the end-node's most previous branchpoint. As an example, a migration starting from “SP” occurs somewhere on the branch between “SP” and the most recent common ancestor of “SP” and “VA.”

**Table 2:** Significant *F*3 Statistics

Pop(A;B,C)	*F*3	SE	*Z* score
AR; CH, SP	−0.00165	0.000171	−9.63768
AR; CR, SP	−0.00178	0.000171	−10.4413
AR; CN, SP	−0.00078	0.00015	−5.15567
AR; OV, SP	−0.00082	0.000145	−5.63104
AR; RO, SP	−0.00076	0.000151	−5.05319
AR; SE, SP	−0.00077	0.00014	−5.47246
LC; CH, SP	−0.00146	0.000193	−7.56011
LC; CR, SP	−0.00154	0.000211	−7.31075
LC; OV, SP	−0.00054	0.00018	−3.00356
RI; CH, SP	−0.00285	0.000948	−3.00128
RI; CR, SP	−0.00296	0.000965	−3.06668

Note.—Population comparisons for which *F*3 statistics were significantly negative (*Z* score ≤ 3) are reported, indicating that population A contains admixture from populations B and C.

In addition to admixture in RI, Treemix and *F*3 statistics also detected admixture in AR (Chile) from the Spanish population. At *m* = 1, 2, 3, and 4, Treemix detected a migration edge from the Spanish branch to AR with migration weight varying between 9% and 17%. *F*3 statistics of AR and Spain with any population from North or Central resulted in a significantly negative value, providing strong evidence of a migration event from a Spanish ancestor like the signal detected with RI. This suggests that the admixture signal we see in AR is from the same Chilean population that gave rise to the Spanish invasion and RI admixture.

### Divergence of *T. absoluta* Predates Modern Tomato Agriculture

**Fig. 4.**
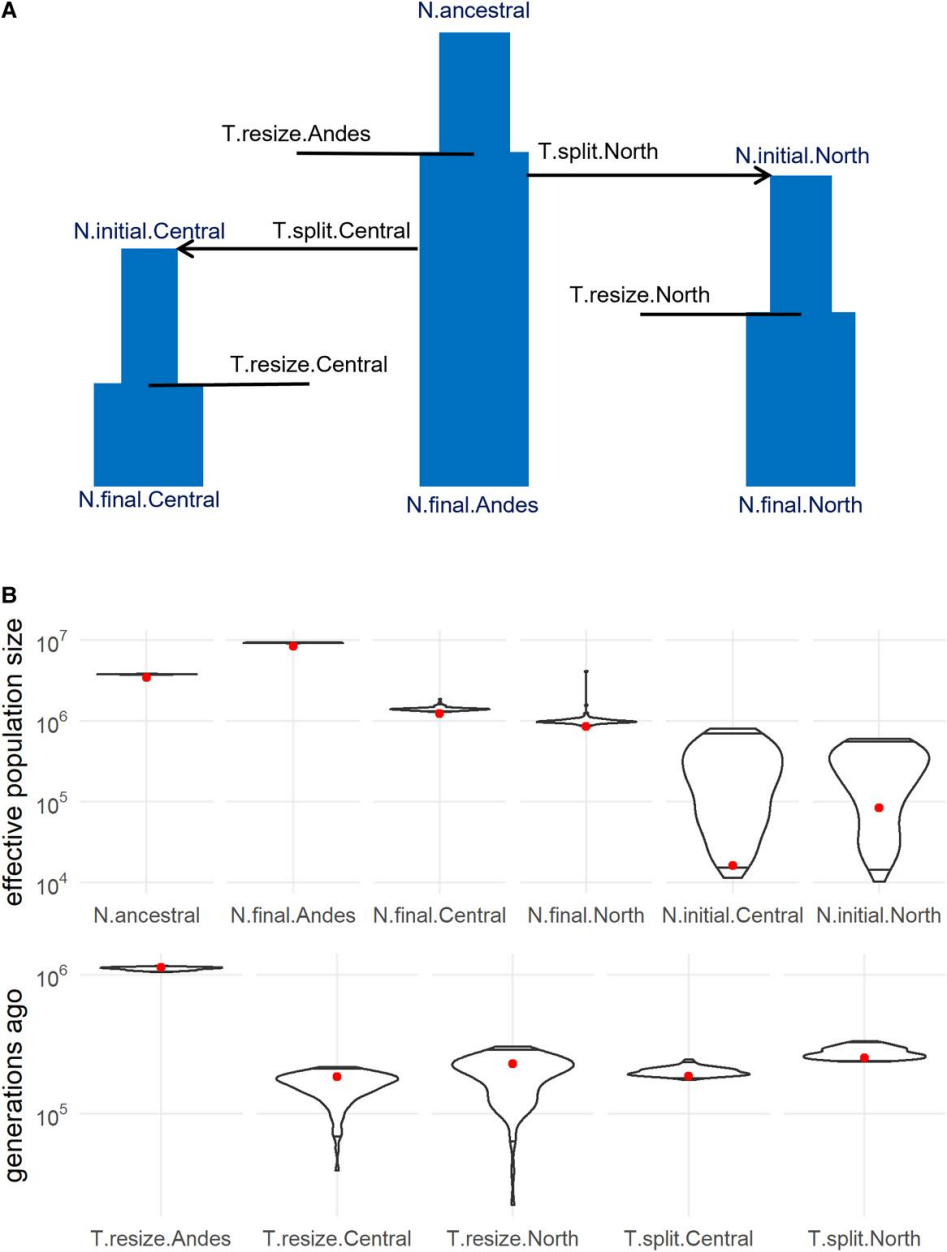
Coalescent model and best estimated parameter values for *T. absoluta* in Latin America. (*A*) Model for a three-population split with population resizing events for each population. (*B*) Maximum likelihood estimates of each parameter in the model indicated by a single dot. Violin plot depicts distribution of parameter values from 100 parametric bootstraps, with upper and lower boundary lines indicating the 95% interval. All point estimates were within the 95% bootstrap intervals except N.ancestral, N.final.Andes, N.final.Central, and N.final.North.

Based on the high levels of nucleotide diversity and *F*st between the Andes, Central, and North clusters, we hypothesized that the three regions may have diverged many generations ago, before the appearance and detection of *T. absoluta* in agricultural crops throughout South America in the mid-20th century. This would suggest a model in which *T. absoluta* transitioned from local, wild host plants to nearby tomato fields independently, rather than a single population being spread to tomato fields through human transport in the 20th century. To investigate this, we calculated the folded two-dimensional site frequency spectrum (2D-SFS) between populations and estimated parameter values under various population models using ML coalescent methods (supplementary fig. S6 and Table S2, Supplementary Material online). We excluded the VA samples from the Andes cluster to avoid potential modeling issues due to VA appearing to originate from a distinct ancestor than other Andes populations. The simplest model allows for two population splits with constant population sizes, while the exponential growth model adds an exponential growth rate to each population. As exponential growth may not be appropriate if divergence times are long, we also tested a model with a simple resizing event for each population at some point in time. We used a post hoc comparison of simulated linkage disequilibrium (LD) decay rates between models to test model fit. We found that while all three models simulated decay rates within the 95% confidence interval (CI) of the Andes population data, none simulated decay rates that overlapped with Central and North decay rate estimates, although the resizing population model was closest (supplementary fig. S7, Supplementary Material online). The lack of fit suggests that there are additional complex historical events not well captured in these models. Under the resizing population model, divergence of the North occurred 252,383 generations ago (95% CI: 243,535–326,583), followed by a Central population divergence 187,034 generations ago (95% CI: 181,668–235,424) (fig. 4; supplementary table S2, Supplementary Material online). Reports of *T. absoluta* generation times can be as high as 6–12 or more generations per year (EPPO 2005; Mansour et al. 2018; de Campos et al. 2021), dating these divergence events to tens of thousands of years ago. This suggests that *T. absoluta* was already present across Latin America prior to the 1960s and, as tomato agriculture surged, transitioned to the new host plant.

### PBS Screening Identifies Several Peaks under Selection

**Fig. 5.**
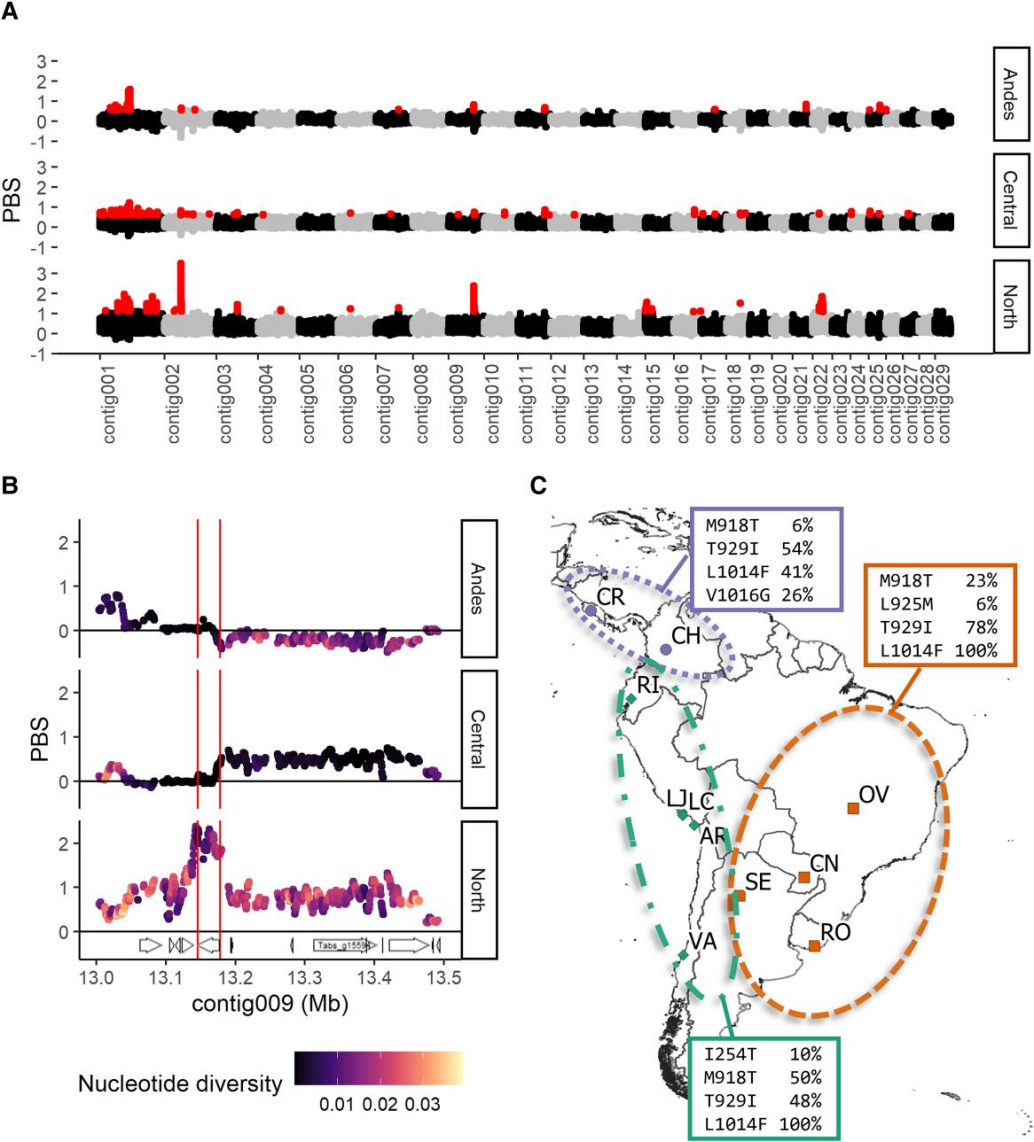
Selection signals in *T. absoluta* based on PBS detect insecticide resistance alleles. (*A*) PBS values in each region calculated across the largest 29 contigs in 5-kb intervals with 500-bp steps. Red points indicate the highest 0.1% PBS values. (*B*) Plot of PBS and genetic diversity (pi) at contig009. Vertical lines bracket the PARA gene model position. (*C*) Map of sampling locations with allele frequencies of known amino acid substitutions in PARA that confer pyrethroid resistance within each cluster. Note the L1014*F* allele, which was found in all Central and Andes samples, while at 41% in North samples.

The Population Branch Statistic (PBS) is an *F*st-based statistic that uses *F*st data between three populations to calculate the population-specific allele frequency changes. Regions of the genome with abnormally high PBS may be under strong selective forces, causing the loci allele frequencies to change faster than expected by drift. We calculated PBS across the genome for all three populations and found several peaks in contigs 2, 9, 15, and 22 that were exceptionally high and broad, particularly in the North cluster (fig. 5*A*). The peak in contig 9 contained the gene *paralytic* (*para*, *T. absoluta* gene g15590), a neuronal sodium channel protein that is the active target of dichlorodiphenyltrichloroethane (DDT) and pyrethroid insecticides (Dong et al. 2014). While PBS peaks in the North population between 13.1 and 13.2Mb on contig009, we note that the allelic diversity was low in the Andes and Central clusters relative to the North (fig. 5*B*). We calculated allele frequencies of known resistance-inducing mutations in each cluster (Dong et al. 2014). Notably, we found that the mutation known as the “kdr” or “knockdown resistance” allele (Guerrero et al. 1997), an alanine to leucine substitution at position 1014 (numbered using the *Musca domestica* gene as a reference), was fixed in the Central and Andes, while at 41% frequency in the North (fig. 5*C*). In addition, we found low to intermediate frequencies of other resistance alleles, including M918T, which in combination with L1014F is known as the “super-kdr” allele (Guerrero et al. 1997), as well as T929I, V1016G, L925M, and I254T.

A similar selective sweep signal was also seen in the PBS hot spot on contig 2 (8.54–8.58 Mb), with high PBS and diversity levels in the North and a large region of low allelic diversity in the Andes and Central (supplementary fig. S8*A*, Supplementary Material online). Several genes were captured in this interval, including *NADH:Ubiquinone oxidoreductase subunit A8* (*ndufa8*), *mucin-5AC-like*, a gene putatively related to human *mucin-5AC*, and two hemomucin genes involved in hemocyte adhesion and innate immunity in insects. The PBS hot spot on contig 15 contained *cryptochrome-2* (*cry2*), encoding a key component of the circadian clock (supplementary fig. S8*B*, Supplementary Material online). Finally, the large hot spot on contig 22 contained 69 genes, including multiple copies of *juvenile hormone binding protein*, ribosomal proteins, gustatory response genes, rhodopsins, and *acetylcholinesterase* (*ache*, also known as *ace* or *ace-1*), a gene implicated in organophosphate insecticide resistance (supplementary fig. S8*C*, Supplementary Material online). Interestingly, based on alignment data and site allele frequency (SAF) estimates output by analysis of next generation sequencing data (ANGSD), it appears that the Central and Andes populations may have two copies of *ache*, while North populations only have one. We looked for known mutations conferring organophosphate resistance and found moderate frequencies in all three clusters as well (supplementary table S3, Supplementary Material online).

## Discussion

Using whole-genome sequencing data, we found that *T. absoluta* samples collected from 11 locations in Latin America clustered into three basic regions, comprised of a North, Andes, and Central group. In addition, we see that Spanish populations likely originated from a Central Chilean source based on their low level of *F*st with the Andes and location on the ML tree. Previous analyses with mitochondrial sequences were unable to differentiate populations (Cifuentes et al. 2011); however, analyses using microsatellite data were able to identify these same three clusters and suggest a Central Chilean source for the European migration as well (Guillemaud et al. 2015). In agreement with this conclusion, looking at fresh tomato export data, we see that Chile is a worldwide exporter, shipping 12 tons of tomatoes an average distance of 11,700 km in 2018, while most other countries in South America tend to export within the continent (FAO 2018).

The Andes Mountains represent an obvious geographic barrier that would separate the Central population from the North and Andes populations. Population structure between Andes and North populations may be due to factors related to the changing latitude, including temperature and day length. While PCA groups our Ecuador samples (RI) with Andes populations, both admixture analysis and Treemix provided some evidence that Ecuador may represent an admixture zone between the two regions. The weighted *F*st between the North and Andes is also lower than between North and Central, suggesting that North and Andes are indeed more closely related. This general *F*st pattern was also observed based on microsatellite analyses (Guillemaud et al. 2015). Sequencing of more samples from Peru and Ecuador might be needed to further elucidate the extent of an admixture zone between these clusters.

While *T. absoluta* was first discovered in Peru in 1917, its native range is not well established. One hypothesis is that *T. absoluta* migrated out of the Andes region and across South America through the 1960s to 1980s because of human transport by agricultural shipping. This aligns with the surge in domestic tomato agriculture in South America at the same time (Minami 1980). However, based on the similar nucleotide diversity levels between clusters, as well as high levels of *F*st, we hypothesized that it might be more likely that this migration across South America may have happened prior to tomato commercialization, with populations of *T. absoluta* transitioning to commercial tomato fields nearby. Based on our simple three population model, it appears that the ancestral population diverged twice tens of thousands of years ago. Relative to the ancestral population size, the combined effective population size is roughly three times larger, although this is heavily weighted toward a very large Andes population, relative to the North and Central regions.

The fact that the estimated Andes population size is nearly ten times larger than that of Central or North populations, as well as its slightly higher level of genetic diversity, could suggest that the Andes cluster represents the ancestral population range. Given that the wild ancestors of tomatoes and potatoes are also native to the Andes region (Spooner et al. 2005; Peralta and Spooner 2006), the Andes region would be an ideal place to search for native parasitoids for biocontrol. To date, biocontrol methods in South America have relied on nonnatives or generalist parasitoids, with only a handful of native specialists identified in the literature and none that are commercially available (Salas Gervassio et al. 2019; Desneux et al. 2022). Thus, knowledge of *T. absoluta*'s native range may help focus efforts to identify more natural parasitoids.

Using the PBS, we found multiple genomic windows under apparent selective forces. Not surprisingly, one of the highest PBS windows contained the *para* gene, which encodes a sodium ion channel that is targeted by pyrethroids. The extremely low allele diversity in the Andes and Central populations relative to the North suggests a hard selective sweep occurred here. Heavy pyrethroid use in Brazil led to the appearance of resistant strains starting in the 1990s (Siqueira et al. 2000). We found that Central and Andes populations were completely fixed for the L1014F mutation, one of the most common causes of kdr to pyrethroids (Dong et al. 2014), while the North had an intermediate frequency. A study looking at Brazilian populations found a similar pattern of fixed L1014 (Silva et al. 2015), while another study looking at multiple populations in South America also found the same pattern of L1014F fixation in Central and Andes populations but not in the North (Haddi et al. 2012). Although both studies also found M918T and T929I at elevated frequencies in all populations, we additionally detected the resistance allele V1016G in the North. We also found L925M in Central and I254T in Andes. Although these two mutations have not been characterized as resistance alleles, mutations at orthologous positions have been shown to confer resistance in *Drosophila melanogaster* (I254N) and *Bemisia tabaci* (L925I) (Pittendrigh et al. 1997; Morin et al. 2002). Further investigation is warranted to determine whether these new alleles also confer insecticide resistance.

The elevated PBS region in contig 22 was relatively large at ∼1 Mb in size, containing over 60 genes. Within this region was the gene *ache*, which encodes a protein that degrades the neurotransmitter acetylcholine (Mutero et al. 1994). As this enzyme is the main target of organophosphates and carbamate insecticides, mutations in *ache* have been associated with resistance. Using the amino acid numbering scheme based on *Torpedo californica* (Massoulié et al. 1992), the resistance allele A201S has previously been reported to be present in European populations (Haddi et al. 2017), and we found this allele to be at moderate to high frequency in all three regions. We also found moderate frequencies of the mutation F290V and F290N. The F290V resistance allele has been documented in mosquitoes and moths (Cassanelli et al. 2006; Alout et al. 2009; Carvalho et al. 2013), while other mutations such as F290Y have been documented in *D. melanogaster* and *M. domestica* (Mutero et al. 1994; Walsh et al. 2001). Interestingly, we noticed two copies of *ache* in the reference genome. We checked for presence of this duplication in our Latin American populations by looking at the number of mapped reads and by looking at the SAF estimates output by ANGSD. We found that although the Central and Andes populations appeared to also have two copies, the North population only contained a single copy of *ache*. Duplication of the *ache* gene has been documented in several insect species and is believed to reduce the heavy selection cost of these resistance mutations by allowing the expression of both wild-type and resistant alleles (Alout et al. 2009; Sonoda et al. 2014). This large structural duplication is likely advantageous and may be the reason for elevated PBS levels across such a large genomic interval. Follow-up work with long-read methods or higher sequencing coverage will be needed to confirm the presence of structural duplication at this locus in different Latin American populations. Additionally, as we did not explicitly test resistance levels to pyrethroids or organophosphates in the sampled populations, further experimental studies will be needed to confirm our genomic findings.

Other regions under selection were less obvious to characterize. We found one region in contig 2 containing *Ndufa8* and several hemomucin/mucin genes with a low genetic diversity in the Central and Andes populations and high PBS in North, indicating a hard selective sweep. *Ndufa8* produces a nuclear-encoded subunit of the NADH dehydrogenase complex I, part of the electron transport chain in the mitochondria used to generate ATP. Mutations here are known to cause mitochondrial complex I deficiency in humans (Yatsuka et al. 2020), although a few studies have found evidence of positive selection occurring in other species, potentially related to metabolism (Kozell et al. 2020; Lee et al. 2020). The hemomucin genes are a component of the insect immune system that are involved in endocytosis (Schmidt et al. 2010). Selection here could be in response to the increased use of parasitoids and predators such as *Trichogramma evanescens* and *Nesidiocoris tenuis* as a biological control alternative to insecticides (Han et al. 2019). We also found evidence of selection based on the PBS on contig 15 near the *cry-2* gene, an important component of the molecular circadian clock. Selection studies in natural populations of several insect species have found evidence of selection in other clock genes as well and are often attributed to selection in facultative diapause (Pegoraro et al. 2017; Zhang et al. 2022). Given that diapause has been detected in French *T. absoluta* populations, it is possible that selection for facultative diapause is occurring in Latin America as well (de Campos et al. 2021). Further analysis will be needed to identify mutations responsible for this selection signal and relevant phenotypes in *T. absoluta*.

We expect that addition of a new contiguous genome assembly with annotations will be of benefit to the *T. absoluta* and Lepidopteran research community. Previous studies have worked to develop potential RNA interference (RNAi) strategies to use as an alternative to traditional pesticides (De Camargo et al. 2015). Work is also being conducted to develop Cas9 gene-editing techniques for *T. absoluta* to facilitate future genetics studies (Ji et al. 2022). These developments in combination with an accurate assembly and gene annotations will allow for accelerated research toward understanding *T. absoluta* biology and methods to contain its economic impacts and spread.

## Methods

### High Molecular Weight DNA Extraction

For genome assembly, a single *T. absoluta* larva was collected in 2021 from a colony originally sourced from the IRTA, Cabrils, Spain, and held in the Contained Research Facility in UC Davis and frozen on dry ice. The larva was pulverized in liquid nitrogen with a pestle in a 2-mL microcentrifuge tube using 740 mL of lysis buffer (80 mm EDTA pH 8, 324 mm NaCl, 0.68% SDS, 8 mm Tris–HCl pH 8, 80 *µ*g/mL RNase A (NEB, Ipswich, MA, USA), 135 *µ*g/mL Prot K[NEB]). After a 37 °C overnight incubation step, 240 *µ*L of 5 m NaCl was added and gently mixed in by rocking before centrifuging at 10,000 RCF, 4 °C, for 15 min. Supernatant was transferred using a wide-bore pipette to a 2-mL DNA low-bind tube (Eppendorf, Enfield, CT, USA), precipitated with 1 mL of 100% ethanol, and centrifuged at 10,000 RCF, 4 °C, for 5 min. The DNA pellet was washed with 500 *µ*L of ice-cold 70% ethanol twice before air-drying for 5 min. Dry pellet was resuspended in diethyl pyrocarbonate (DEPC)-treated water and allowed to dissolve for 1 h at room temperature before being stored at 4 °C for no more than 2 weeks. Absorbance ratios were measured with a NanoDrop Lite (Thermo Fisher Scientific, Waltham, MA, USA), DNA concentration was measured with a Qubit 4 Fluorometer using a dsDNA High-Sensitivity Assay (Thermo Fisher Scientific), and DNA fragment size was measured with a TapeStation genomic DNA ScreenTape (Agilent, Santa Clara, CA, USA). Approximately 700 ng of DNA was sent to QB3-Berkeley for library preparation and PacBio HiFi sequencing with 1 SMRTcell.

### Genome Assembly and Assessment

Raw subreads were collapsed into Circular Consensus Sequence (CCS) reads using CCS version 6.0.0 (PacBio, Menlo Park, CA, USA). K-mer histograms were made with jellyfish version 2.2.6 (Marçais and Kingsford 2011) using 31-mers, then visualized with GenomeScope version 2.0 (Ranallo-Benavidez et al. 2020). GC content versus k-mer frequency was calculated from the jellyfish histograms using kat version 2.4.2 (Mapleson et al. 2016) and visualized with R.

CCS reads were initially assembled using hifiasm version 0.14 or HiCanu version 2.11 (Nurk et al. 2020; Cheng et al. 2021) with default parameters. HiCanu assembly was separated into a primary and alternate haplotig set using purge-dups version 1.2.5 4 (Guan 2021). The primary hifiasm assembly was purged with purge_dups; alternate haplotigs from this purge were purged with the alternate hifiasm assembly and repurged with purge_dups to discard repeats, high-coverage, or nested haplotigs.

Merqury version 1.1 (Rhie et al. 2020) was used to assess genome assembly quality and completeness between the two assemblers and between prepurging and postpurging. K-mer size of 20 was used for building the Meryl database from the raw CCS reads. Copy number k-mer plots were generated using Merqury's provided R scripts. BUSCO version 5.1.2 (Manni et al. 2021) was also used in genome mode to assess genome ortholog completeness using the Lepidoptera OrthoDB-10 database (Kriventseva et al. 2019).

To detect contigs that were contaminant DNA and not of *T. absoluta* origin, BlastN version 2.12.0 (Camacho et al. 2009) was used with the “nt” database (downloaded August 3rd, 2021) under the following parameters: word size = 20 and max target sequences = 10. Taxonomic information was downloaded for each subject match from the NCBI Taxonomy database using the “rentrez” package in R. To corroborate Blast results, Phyloligo version 1.0 (Mallet et al. 2017) was used to generate a Euclidian distance matrix between contigs based on k-mer distribution with k-mer length 4. The R packages “ape” version 5.6–1 (Paradis and Schliep 2019) and “ggtree” version 2.2.4 (Yu et al. 2017) were used to generate and visualize the contig tree using the BIONJ algorithm.

### Genome Annotation

#### Repeat Masking

The decontaminated hifiasm primary genome assembly was supplied as a database to RepeatModeler version 2.0.2a (Flynn et al. 2020) to produce a custom repeat library, with the long terminal repeat module enabled. RepeatMasker version 4.1.2 (Smit et al. 2021) was used to produce GFF annotation files of repeat coordinates. The Dfam transposable element database provided with RepeatMasker was merged with the RepBase RepeatMasker Edition database version 20181026 (Bao et al. 2015) to mask the genome once, and the custom RepeatModeler library was used to mask the genome separately. The resulting GFF files were merged and sorted with bedtools version 2.30 sort (Quinlan and Hall 2010) and then used to soft-mask the assembly with bedtools maskfasta.

#### Gene Model Annotation

Six RNAseq data sets produced by De Camargo et al. (2015) covering all life stages of *T. absoluta* (egg, four larval instars, and adult) were downloaded from Bioproject PRJNA291932 for gene model annotation. Reads were checked for quality with FastQC version 0.11.9 (Babraham Bioinformatics 2019), trimmed with Trimmomatic version 0.39 (Bolger et al. 2014), and aligned to our primary assembly using STAR version 2.7.9a (Dobin et al. 2013) with default parameters. In addition, protein databases were downloaded from Lepbase (Challi et al. 2016) and OrthoDB-arthropoda (Kriventseva et al. 2019). The soft-masked genome was annotated twice with BRAKER2 version 2.1.5 (Brůna et al. 2021), once with the aligned RNA data, and again with the merged protein data. The two resulting GTF gene model files, as well as the GFF gene model hints files, were supplied to TSEBRA version 1.0.2 (Gabriel et al. 2021) to be merged into a single GTF output. Gffread version 0.12.6 (Pertea and Pertea 2020) was used to remove mRNAs with missing start or stop codons, in-frame stop codons, or those that were redundant.

#### Functional Gene Annotation

Entap version 0.10.7 (Hart et al. 2020) was used to annotate gene models with names and predicted functions. Entap was configured to perform frame selection and filtering. The Lepbase, Refseq Invertebrate, UniprotKB/Swiss-Prot, and UniprotKB/TrEMBL (Challi et al. 2016; O’Leary et al. 2016; The UniProt Consortium 2021) protein databases were used for gene identity search and the EggNOG database (Huerta-Cepas et al. 2019) for gene ontology, protein domain, and pathway annotation. As EnTAP was designed for transcriptome annotation, gene model coordinates were not referenced to the genome assembly. To correct this, the output GFF gene model annotation was converted to a GTF using gffreads, then converted to an alignment-GFF format using the script “gtf_to_alignment_gff3.pl” from Transdecoder (Brian and Papanicolaou), and finally mapped back to the genome assembly coordinates using the Transdecoder script “cdna_alignment_orf_to_genome_orf.pl.”

### Population Sample DNA Extraction and Alignment

We used the same DNA extracted from *T. absoluta* collected from South America, CR, and Spain by Tabuloc et al. (2019). All samples were collected in 2017. DNA libraries were made using the KAPA Hyperplus Kit (Roche, Basel, Switzerland). A 150-bp paired-end sequencing was performed by Novogene on the Illumina HiSeq 4000. Raw reads were trimmed of adapter sequences using scythe version 0.991 (Buffalo 2014) and were quality-filtered using sickle version 1.33 (Joshi and Fass 2011) using default settings. FastQC was used to inspect read quality before and after filtering. Reads were mapped to the genome assembly using bwa mem version 0.7.17 (Li 2013), and duplicates were marked with samtools markdup version 1.14 (Danecek et al. 2021).

### Population Structure Analysis

Angsd version 0.935 (Korneliussen et al. 2014) was used to estimate genotype likelihoods from the 78 contigs longer than 100 kb, using a SNP filter threshold of p < 10^−6^, a minimum minor allele frequency of 0.05, and a minimum map and base quality of Q = 20. SNPs were then pruned to every 500 bp. PCA and admixture analysis were performed using PCAngsd version 1.0 (Meisner and Albrechtsen 2018) and NGSadmix version 32 (Skotte et al. 2013) as described in Lewald et al. 2021. PCAngsd was also used to output inbreeding coefficients for each sample.

### Treemix

We called genotypes from the genotype likelihoods calculated for population structure analysis using PCAngsd, with a 95% confidence threshold and with inbreeding values estimated from PCAngsd as priors. We removed loci that were missing data in more than 20% of samples, or loci with missing data in all individuals within a single sampling location. A custom R script was used to convert PCAngsd-format genotypes into a Treemix-formatted allele counts table. Treemix version 1.13 (Pickrell and Pritchard 2012) was run with 100 bootstraps, a window block size of 500 SNPs and 0 to 5 migration edges. The “global rearrangements” and “standard error calculation” options were also enabled. The CR population was used to root the tree, as the North region was most divergent relative to all other regions based on *F*st values (supplementary fig. S5, Supplementary Material online). Treemix's “threepop” subprogram was used to calculate *F*3 statistics between populations, using a 500 SNP window block size for standard error estimation.

### Population Summary Statistics and PBS

Summary statistics were calculated with Angsd on the largest 78 contigs. The SAFs were calculated for each region (North, Andes, Central, and Spain) with the -doMaf 1 option and using individual inbreeding coefficients as priors, and no minor allele frequency or SNP filtering was used. To estimate nucleotide diversity and Tajima's D, the global folded one-dimensional site frequency spectrum (1D-SFS) was calculated using Angsd realSFS for each population using the SAF in 100-Mb pieces of the genome with a maximum of 400 iterations in the EM cycle. The 1D-SFS was summed across the genome for each population, and realSFS saf2theta was used to estimate thetas per site. Angsd thetaStat do_stat was then used to calculate theta and Tajima's D in 20-kb windows, with a step of either 20 or 5 kb.

To estimate *F*st between regions, realSFS was used to calculate the global folded 2D-SFS between each pair of regions in 100Mb pieces of the genome with 400 EM cycle iterations. 2D-SFS was summed across the genome, and realSFS *F*st index was then used to estimate per-site *F*st, and realSFS fst stats was used to estimate the global *F*st values between regions. Weighted *F*st was used for comparisons as it is less biased when using many rare, population-specific SNPs (as is the case when genotyping by whole-genome sequencing) (Bhatia et al. 2013). To estimate the PBS, the Andes, Central, and North regions were supplied at once to realSFS *F*st index and realSFS fst stats2, to produce PBS values for each region in sliding 5-kb windows with a 500-bp step across the genome. Windows with <4 kb of sequence data were excluded from the analysis. To compare allele frequencies of key SNPs between populations, we repeated SAF estimation but forced the reference allele to be the “major” allele.

### Population Modeling

The 2D-SFS was estimated between North, Andes, and Central regions using the same procedure as for summary statistics but excluded all genic regions (gene model boundaries plus an additional 1 kb on flanking sides). Additionally, the VA, Chile, population samples were excluded from the analysis. Output 2D-SFS was converted to the Fastsimcoal version 2708 (Excoffier et al. 2013) format using a custom R script.

Fastsimcoal was used to estimate model parameters from the 2D-SFS data under several possible models. In the simplest model, an ancestral population is allowed to split two times, each with its own population size (five total parameters). The exponential growth model adds a growth event to each population with unique exponential growth rates (nine total parameters). Finally, the resized population model replaces the exponential growth with an instantaneous change in population size (11 total parameters). For each model, parameter estimation was run 100 independent times, using 1 million simulations and 100 EM loops per run. SFS categories with less than ten counts were excluded.

To compare models to each other and the data, 1Mb of DNA was simulated 100 times under each model using Fastsimcoal with a mutation rate of 2.9 × 10^−9^ bp/generation (Keightley et al. 2015) and a recombination rate of 2.97 × 10^−8 ^cM/Mb (Yamamoto et al. 2008), based on estimates from *Heliconius melpomene* and *Bombyx mori*, respectively. The resulting genotypes were used to estimate the *r*^2^ ­measure of LD between SNPs <10 kb away using ngsLD version 1.1.1 (Fox et al. 2019). To estimate LD from the sequencing data, the same minor allele frequency files used for Fastsimcoal parameter estimation were used to call genotype likelihoods in each region on the largest 29 contigs. NgsLD was run on a 10% subset of these genotype likelihoods to a max distance of 10 kb. Estimates of LD decay rates, maximum, and minimum LD were calculated from a 1% subset of *r*^2^ values from simulations and a 10% subset of *r*^2^ values from data using the provided fit_LDdecay.R script with the following parameters: fit_bin_size = 100, recombination rate = 2.97, and fit_boot = 100, fit_level = 10.

## Supplementary Material

evad060_Supplementary_Data

## Data Availability

Scripts used for genome assembly and population analysis are available at github.com/ClockLabX/Tabs-genome-popgen. Genome assembly and Latin American population raw sequences are available on the NCBI BioProject database under BioProject ID# PRJNA909831. Raw sequences of Spanish *T. absoluta* individuals are available under BioProject ID# PRJNA512383. Additional supporting data including RepeatMasker and EnTAP annotations, allele frequencies, and PBS values are available on Data Dryad at https://doi.org/10.25338/B8XD1C.

## References

[evad060-B1] Alout H , Labbé P, Berthomieu A, Pasteur N, Weill M. 2009. Multiple duplications of the rare ace-1 mutation F290V in *Culex pipiens* natural populations. Insect Biochem Mol Biol. 39:884–891. 19874892 10.1016/j.ibmb.2009.10.005

[evad060-B2] Babraham Bioinformatics . 2019. FastQC. https://www.bioinformatics.babraham.ac.uk/projects/fastqc/.

[evad060-B3] Bao W , Kojima KK, Kohany O. 2015. Repbase update, a database of repetitive elements in eukaryotic genomes. Mob DNA. 6:11. 26045719 10.1186/s13100-015-0041-9 PMC4455052

[evad060-B4] Bhatia G , Patterson N, Sankararaman S, Price AL. 2013. Estimating and interpreting FST: the impact of rare variants. Genome Res. 23:1514–1521. 23861382 10.1101/gr.154831.113 PMC3759727

[evad060-B5] Bolger AM , Lohse M, Usadel B. 2014. Trimmomatic: a flexible trimmer for Illumina sequence data. Bioinforma Oxf. Engl. 30:2114–2120. 10.1093/bioinformatics/btu170 PMC4103590 24695404

[evad060-B6] Brian H , Papanicolaou A. TransDecoder. http://transdecoder.github.io.

[evad060-B7] Brůna T , Hoff KJ, Lomsadze A, Stanke M, Borodovsky M. 2021. BRAKER2: automatic eukaryotic genome annotation with GeneMark-EP+ and AUGUSTUS supported by a protein database. NAR Genomics Bioinforma 3:lqaa108. 10.1093/nargab/lqaa108 PMC7787252 33575650

[evad060-B8] Buffalo V. 2014. Scythe: A 3'-end adapter contaminant trimmer. https://github.com/vsbuffalo/scythe.

[evad060-B9] Camacho C , et al 2009. Blast+: architecture and applications. BMC Bioinformatics 10:421. 20003500 10.1186/1471-2105-10-421 PMC2803857

[evad060-B10] Carvalho RA , Omoto C, Field LM, Williamson MS, Bass C. 2013. Investigating the molecular mechanisms of organophosphate and pyrethroid resistance in the fall armyworm *Spodoptera frugiperda*. PLoS One 8:e62268. 10.1371/journal.pone.0062268 PMC3629120 23614047

[evad060-B11] Cassanelli S , Reyes M, Rault M, Carlo Manicardi G, Sauphanor B. 2006. Acetylcholinesterase mutation in an insecticide-resistant population of the codling moth *Cydia pomonella* (L). Insect Biochem Mol Biol. 36:642–653. 16876707 10.1016/j.ibmb.2006.05.007

[evad060-B12] Challi RJ , Kumar S, Dasmahapatra KK, Jiggins CD, Blaxter M. 2016. Lepbase: the Lepidopteran genome database. bioRxiv:056994.

[evad060-B13] Chang PEC , Metz MA. 2021. Classification of *Tuta absoluta* (Meyrick, 1917) (Lepidoptera: Gelechiidae: Gelechiinae: Gnorimoschemini) based on cladistic analysis of morphology. Proc Entomol Soc Wash. 123:41–54.

[evad060-B14] Cheng H , Concepcion GT, Feng X, Zhang H, Li H. 2021. Haplotype-resolved de novo assembly using phased assembly graphs with hifiasm. Nat Methods. 18:170–175. 33526886 10.1038/s41592-020-01056-5 PMC7961889

[evad060-B15] Cifuentes D , Chynoweth R, Bielza P. 2011. Genetic study of Mediterranean and South American populations of tomato leafminer *Tuta absoluta* (Povolny, 1994) (Lepidoptera: Gelechiidae) using ribosomal and mitochondrial markers. Pest Manag Sci. 67:1155–1162. 21495155 10.1002/ps.2166

[evad060-B16] Danecek P , et al 2021. Twelve years of SAMtools and BCFtools. GigaScience 10:giab008. 10.1093/gigascience/giab008 PMC7931819 33590861

[evad060-B17] De Camargo RA , et al 2015. De novo transcriptome assembly and analysis to identify potential gene targets for RNAi-mediated control of the tomato leafminer (*Tuta absoluta*). BMC Genomics 16:635.. 26306628 10.1186/s12864-015-1841-5 PMC4550053

[evad060-B18] de Campos MR , et al 2021. Thermal biology of *Tuta absoluta*: demographic parameters and facultative diapause. J Pest Sci. 94:829–842.

[evad060-B19] Desneux N , et al 2022. Integrated pest management of *Tuta absoluta*: practical implementations across different world regions. J Pest Sci. 95:17–39.

[evad060-B20] Desneux N , Luna MG, Guillemaud T, Urbaneja A. 2011. The invasive South American tomato pinworm, *Tuta absoluta*, continues to spread in Afro-Eurasia and beyond: the new threat to tomato world production. J. Pest Sci. 84:403–408.

[evad060-B21] Dobin A , et al 2013. STAR: ultrafast universal RNA-seq aligner. Bioinformatics 29:15–21. 23104886 10.1093/bioinformatics/bts635 PMC3530905

[evad060-B22] Dong K , et al 2014. Molecular biology of insect sodium channels and pyrethroid resistance. Insect Biochem Mol Biol. 50:1–17. 24704279 10.1016/j.ibmb.2014.03.012 PMC4484874

[evad060-B23] EPPO . 2005. Tuta absoluta. EPPO Bull. 35:434–435.

[evad060-B24] EPPO . 2008. First record of *Tuta absoluta* in Spain. EPPO Report Serv.

[evad060-B25] Excoffier L , Dupanloup I, Huerta-Sánchez E, Sousa VC, Foll M. 2013. Robust demographic inference from genomic and SNP data. PLOS Genet. 9:e1003905. 10.1371/journal.pgen.1003905 PMC3812088 24204310

[evad060-B26] FAOSTAT . 2020. Worldwide Production of Tomatoes. Food Agric. Organ. U. N. http://www.fao.org/faostat (Accessed October 18, 2022).

[evad060-B27] Flynn JM , et al 2020. Repeatmodeler2 for automated genomic discovery of transposable element families. Proc Natl Acad Sci U S A. 117:9451–9457. 32300014 10.1073/pnas.1921046117 PMC7196820

[evad060-B28] Fox EA , Wright AE, Fumagalli M, Vieira FG. 2019. ngsLD: evaluating linkage disequilibrium using genotype likelihoods. Bioinformatics 35:3855–3856. 30903149 10.1093/bioinformatics/btz200

[evad060-B29] Gabriel L , Hoff KJ, Brůna T, Borodovsky M, Stanke M. 2021. TSEBRA: transcript selector for BRAKER. BMC Bioinformatics 22:566. 34823473 10.1186/s12859-021-04482-0 PMC8620231

[evad060-B30] Godfrey K , Zalom F, Chiu J. 2018. Tuta Absoluta, The South American Tomato Leafminer. University of California. Agriculture and Natural Resources.

[evad060-B31] Guan DF. 2021. purge_dups. https://github.com/dfguan/purge_dups.

[evad060-B32] Guerrero FD , Jamroz RC, Kammlah D, Kunz SE. 1997. Toxicological and molecular characterization of pyrethroid-resistant horn flies, *Haematobia irritans*: identification of kdr and super-kdr point mutations. Insect Biochem Mol Biol. 27:745–755. 9443375 10.1016/s0965-1748(97)00057-x

[evad060-B33] Guillemaud T , et al 2015. The tomato borer, *Tuta absoluta*, invading the Mediterranean basin, originates from a single introduction from central Chile. Sci Rep. 5:8371. 25667134 10.1038/srep08371 PMC4322357

[evad060-B34] Haddi K , et al 2012. Identification of mutations associated with pyrethroid resistance in the voltage-gated sodium channel of the tomato leaf miner (*Tuta absoluta*). Insect Biochem Mol Biol. 42:506–513. 22504519 10.1016/j.ibmb.2012.03.008

[evad060-B35] Haddi K , et al 2017. Mutation in the *ace-1* gene of the tomato leaf miner (*Tuta absoluta*) associated with organophosphates resistance. J Appl Entomol. 141:612–619.

[evad060-B36] Han P , et al 2019. *Tuta absoluta* continues to disperse in Asia: damage, ongoing management and future challenges. J Pest Sci. 92:1317–1327.

[evad060-B37] Hart AJ , et al 2020. EnTAP: bringing faster and smarter functional annotation to non-model eukaryotic transcriptomes. Mol Ecol Resour. 20:591–604. 31628884 10.1111/1755-0998.13106

[evad060-B38] Huerta-Cepas J , et al 2019. eggNOG 5.0: a hierarchical, functionally and phylogenetically annotated orthology resource based on 5090 organisms and 2502 viruses. Nucleic Acids Res. 47:D309–D314. 30418610 10.1093/nar/gky1085 PMC6324079

[evad060-B39] Ji S-X , et al 2022. First report on CRISPR/Cas9-based genome editing in the destructive invasive pest *Tuta absoluta* (Meyrick) (Lepidoptera: Gelechiidae). Front Genet. 13:865622. 10.3389/fgene.2022.865622 PMC9160428 35664294

[evad060-B40] Joshi N , Fass J. 2011. Sickle: A sliding-window, adaptive, quality-based trimming tool for FastQ files. https://github.com/najoshi/sickle.

[evad060-B41] Keightley PD , et al 2015. Estimation of the spontaneous mutation rate in *Heliconius melpomene*. Mol Biol Evol. 32:239–243. 25371432 10.1093/molbev/msu302 PMC4271535

[evad060-B42] Koch JB , et al 2020. Population genomic and phenotype diversity of invasive *Drosophila suzukii* in Hawai’i. Biol Invasions. 22:1753–1770.

[evad060-B43] Korneliussen TS , Albrechtsen A, Nielsen R. 2014. ANGSD: analysis of next generation sequencing data. BMC Bioinformatics 15:356. 25420514 10.1186/s12859-014-0356-4 PMC4248462

[evad060-B44] Kozell LB , et al 2020. RNA-Seq Analysis of genetic and transcriptome network effects of dual-trait selection for ethanol preference and withdrawal using SOT and NOT genetic models. Alcohol Clin Exp Res. 44:820–830. 32090358 10.1111/acer.14312 PMC7169974

[evad060-B45] Kriventseva EV , et al 2019. OrthoDB v10: sampling the diversity of animal, plant, fungal, protist, bacterial and viral genomes for evolutionary and functional annotations of orthologs. Nucleic Acids Res. 47:D807–D811. 30395283 10.1093/nar/gky1053 PMC6323947

[evad060-B46] Lee H , et al 2020. Whole genome analysis of the red-crowned crane provides insight into avian longevity. Mol Cells. 43:86–95. 31940721 10.14348/molcells.2019.0190 PMC6999708

[evad060-B47] Lewald KM , et al 2021. Population genomics of *Drosophila suzukii* reveal longitudinal population structure and signals of migrations in and out of the continental United States. G3 11(12):jkab343. 34599814 10.1093/g3journal/jkab343 PMC8664444

[evad060-B48] Li H. 2013. Aligning sequence reads, clone sequences and assembly contigs with BWA-MEM. ArXiv13033997 Q-Bio. http://arxiv.org/abs/1303.3997 (Accessed February 17, 2021).

[evad060-B49] Mackintosh A , et al 2019. The determinants of genetic diversity in butterflies. Nat Commun. 10:3466. 31371715 10.1038/s41467-019-11308-4 PMC6672018

[evad060-B50] Mallet L , Bitard-Feildel T, Cerutti F, Chiapello H. 2017. Phyloligo: a package to identify contaminant or untargeted organism sequences in genome assemblies. Bioinformatics 33:3283–3285. 28637232 10.1093/bioinformatics/btx396 PMC5860033

[evad060-B51] Manni M , Berkeley MR, Seppey M, Simão FA, Zdobnov EM. 2021. BUSCO Update: novel and streamlined workflows along with broader and deeper phylogenetic coverage for scoring of eukaryotic, prokaryotic, and viral genomes kelley, J, editor. Mol Biol Evol. 38:4647–4654. 34320186 10.1093/molbev/msab199 PMC8476166

[evad060-B52] Mansour R , et al 2018. Occurrence, biology, natural enemies and management of *Tuta absoluta* in Africa. Entomol Gen. 38:83–112.

[evad060-B53] Mapleson D , Garcia Accinelli G, Kettleborough G, Wright J, Clavijo BJ. 2016. KAT: a K-mer analysis toolkit to quality control NGS datasets and genome assemblies. Bioinformatics 33(4):574–576. 10.1093/bioinformatics/btw663 PMC5408915 27797770

[evad060-B54] Marçais G , Kingsford C. 2011. A fast, lock-free approach for efficient parallel counting of occurrences of k-mers. Bioinformatics 27:764–770. 21217122 10.1093/bioinformatics/btr011 PMC3051319

[evad060-B55] Massoulié J et al 1992. Recommendations for omenclature in cholinesterases. In: Shafferman, A, Velan B, editors. Multidisciplinary approaches to cholinesterase functions. Springer US: Boston, MA. 285–288. 10.1007/978-1-4615-3046-6_37.

[evad060-B56] Meisner J , Albrechtsen A. 2018. Inferring population structure and admixture proportions in low-depth NGS data. Genetics 210:719–731. 30131346 10.1534/genetics.118.301336 PMC6216594

[evad060-B57] Meyrick E . 1917. I. Descriptions of South American micro-lepidoptera. Trans R Entomol Soc Lond. 65:1–52.

[evad060-B58] Minami K . 1980. The history of tomato production for industry in South America. Acta Hortic 100:87–92.

[evad060-B59] Morin S , et al 2002. Mutations in the *Bemisia tabaci* para sodium channel gene associated with resistance to a pyrethroid plus organophosphate mixture. Insect Biochem Mol Biol. 32:1781–1791. 12429130 10.1016/s0965-1748(02)00137-6

[evad060-B60] Mutero A , Pralavorio M, Bride JM, Fournier D. 1994. Resistance-associated point mutations in insecticide-insensitive acetylcholinesterase. Proc Natl Acad Sci U. S. A. 91:5922–5926. 8016090 10.1073/pnas.91.13.5922 PMC44109

[evad060-B61] Nurk S , et al 2020. Hicanu: accurate assembly of segmental duplications, satellites, and allelic variants from high-fidelity long reads. Genome Res. 30:1291–1305. 32801147 10.1101/gr.263566.120 PMC7545148

[evad060-B62] O’Leary NA , et al 2016. Reference sequence (RefSeq) database at NCBI: current status, taxonomic expansion, and functional annotation. Nucleic Acids Res. 44:D733–D745. 26553804 10.1093/nar/gkv1189 PMC4702849

[evad060-B63] Paladino LZC , et al 2016. The effect of X-rays on cytological traits of *Tuta absoluta* (Lepidoptera: Gelechiidae). Fla Entomol. 99:43–53.

[evad060-B64] Paradis E , Schliep K. 2019. . Ape 5.0: an environment for modern phylogenetics and evolutionary analyses in R. Bioinformatics 35:526–528. 30016406 10.1093/bioinformatics/bty633

[evad060-B65] Pegoraro M , et al 2017. Geographical analysis of diapause inducibility in European *Drosophila melanogaster* populations. J Insect Physiol. 98:238–244. 28131702 10.1016/j.jinsphys.2017.01.015

[evad060-B66] Peralta IE , Spooner DM. 2006. History, origin, and early cultivation of tomato (Solanaceae). Genetic improvement of solanaceous crops vol. 2. Enfield, NH: Tomato Science Publishers. p. 1–24.

[evad060-B67] Pertea G , Pertea M. 2020. GFF utilities: GffRead and GffCompare. F1000Research 9(304). 10.12688/f1000research.23297.1 PMC7222033 32489650

[evad060-B68] Pickrell JK , Pritchard JK. 2012. Inference of population splits and mixtures from genome-wide allele frequency data. PLOS Genet. 8:e1002967. 10.1371/journal.pgen.1002967 PMC3499260 23166502

[evad060-B69] Pittendrigh B , Reenan R, ffrench-Constant RH, Ganetzky B. 1997. Point mutations in the Drosophila sodium channel gene para associated with resistance to DDT and pyrethroid insecticides. Mol Gen Genet. 256:602–610. 9435785 10.1007/s004380050608

[evad060-B70] Quinlan AR , Hall IM. 2010. BEDTools: a flexible suite of utilities for comparing genomic features. Bioinformatics 26:841–842. 20110278 10.1093/bioinformatics/btq033 PMC2832824

[evad060-B71] Ranallo-Benavidez TR , Jaron KS, Schatz MC. 2020. GenomeScope 2.0 and Smudgeplot for reference-free profiling of polyploid genomes. Nat Commun. 11:1432. 32188846 10.1038/s41467-020-14998-3 PMC7080791

[evad060-B72] Rašić G , Filipović I, Weeks AR, Hoffmann AA. 2014. Genome-wide SNPs lead to strong signals of geographic structure and relatedness patterns in the major arbovirus vector, *Aedes aegypti*. BMC Genomics 15:275. 24726019 10.1186/1471-2164-15-275 PMC4023594

[evad060-B73] Reich D , Thangaraj K, Patterson N, Price AL, Singh L. 2009. Reconstructing Indian population history. Nature 461:489. 19779445 10.1038/nature08365 PMC2842210

[evad060-B74] Rhie A , Walenz BP, Koren S, Phillippy AM. 2020. Merqury: reference-free quality, completeness, and phasing assessment for genome assemblies. Genome Biol. 21:245. 32928274 10.1186/s13059-020-02134-9 PMC7488777

[evad060-B75] Salas Gervassio NG , Aquino D, Vallina C, Biondi A, Luna MG. 2019. A re-examination of *Tuta absoluta* parasitoids in South America for optimized biological control. J Pest Sci. 92:1343–1357.

[evad060-B76] Schmidt O , Söderhäll K, Theopold U, Faye I. 2010. Role of adhesion in arthropod immune recognition. Annu Rev Entomol. 55:485–504. 19743913 10.1146/annurev.ento.54.110807.090618

[evad060-B77] Silva WM , et al 2015. Status of pyrethroid resistance and mechanisms in Brazilian populations of *Tuta absoluta*. Pestic Biochem Physiol. 122:8–14. 26071801 10.1016/j.pestbp.2015.01.011

[evad060-B78] Siqueira HÁA , Guedes RNC, Picanço MC. 2000. Insecticide resistance in populations of *Tuta absoluta* (Lepidoptera: Gelechiidae). Agric For Entomol. 2:147–153.

[evad060-B79] Skotte L , Korneliussen TS, Albrechtsen A. 2013. Estimating individual admixture proportions from next generation sequencing data. Genetics 195:693–702. 24026093 10.1534/genetics.113.154138 PMC3813857

[evad060-B80] Smit AFA , Hubley R, Green P. 2021. RepeatMasker. http://repeatmasker.org.

[evad060-B81] Sonoda S , et al 2014. Duplication of acetylcholinesterase gene in diamondback moth strains with different sensitivities to acephate. Insect Biochem Mol Biol. 48:83–90. 24632376 10.1016/j.ibmb.2014.02.008

[evad060-B82] Spooner DM , McLean K, Ramsay G, Waugh R, Bryan GJ. 2005. A single domestication for potato based on multilocus amplified fragment length polymorphism genotyping. Proc Natl Acad Sci U S A. 102:14694–14699. 16203994 10.1073/pnas.0507400102 PMC1253605

[evad060-B83] Tabuloc CA , et al 2019. Sequencing of *Tuta absoluta* genome to develop SNP genotyping assays for species identification. J Pest Sci. 92:1397–1407.

[evad060-B84] Trask JAS , et al 2011. The effect of SNP discovery method and sample size on estimation of population genetic data for Chinese and Indian rhesus macaques (*Macaca mulatta*). Primates 52:129–138. 21207104 10.1007/s10329-010-0232-4

[evad060-B85] The UniProt Consortium . 2021. Uniprot: the universal protein knowledgebase in 2021. Nucleic Acids Res. 49:D480–D489. 33237286 10.1093/nar/gkaa1100 PMC7778908

[evad060-B86] Walsh SB , et al 2001. Identification and characterization of mutations in housefly (*Musca domestica*) acetylcholinesterase involved in insecticide resistance. Biochem J. 359:175–181. 11563981 10.1042/0264-6021:3590175 PMC1222133

[evad060-B87] Willing E-M , Dreyer C, van Oosterhout C. 2012. Estimates of genetic differentiation measured by FST do not necessarily require large sample sizes when using many SNP markers. PLoS One 7:e42649. 10.1371/journal.pone.0042649 PMC3419229 22905157

[evad060-B88] Worldbank . 2006. Spain Vegetables: tomatoes, fresh or chilled imports from Chile in 2006. https://wits.worldbank.org/.

[evad060-B89] FAO . 2018. Worldwide Production of Tomatoes. Food and Agriculture Organization of the United Nations http://www.fao.org/faostat.

[evad060-B90] Yamamoto K , et al 2008. A BAC-based integrated linkage map of the silkworm *Bombyx mori*. Genome Biol. 9:R21. 18226216 10.1186/gb-2008-9-1-r21 PMC2395255

[evad060-B91] Yatsuka Y , et al 2020. A homozygous variant in NDUFA8 is associated with developmental delay, microcephaly, and epilepsy due to mitochondrial complex I deficiency. Clin Genet. 98:155–165. 32385911 10.1111/cge.13773

[evad060-B92] Yu G , Smith DK, Zhu H, Guan Y, Lam TT-Y. 2017. . GGTREE: an R package for visualization and annotation of phylogenetic trees with their covariates and other associated data. Methods Ecol Evol. 8:28–36.

[evad060-B93] Zhang J , et al 2022. Population genomics provides insights into lineage divergence and local adaptation within the cotton bollworm. Mol Ecol Resour. 22:1875–1891. 35007400 10.1111/1755-0998.13581

