# Supplementary material for: Genome Assembly and Population Sequencing Reveal Three Populations and Signatures of Insecticide Resistance of Tuta absoluta in Latin America
Source: Genome Biol Evol. 2023 Apr 18;15(4):evad060. doi: 10.1093/gbe/evad060 (PMC10139443; doi:10.1093/gbe/evad060)

# Supplemental Figures

Figure S1: Summary and quality metrics based on PacBio CCS reads. (A) GenomeScope k-mer profile of PacBio CCS reads, indicating predicted genome length, percent of the genome that is unique, percent homozygosity and heterozygosity, coverage, sequencing error rates, and duplication rates. (B) GC percent vs k-mer frequency plot of CCS reads, excluding k-mers with frequency less than or equal to 5 (to ignore unique k-mers due to sequencing errors).


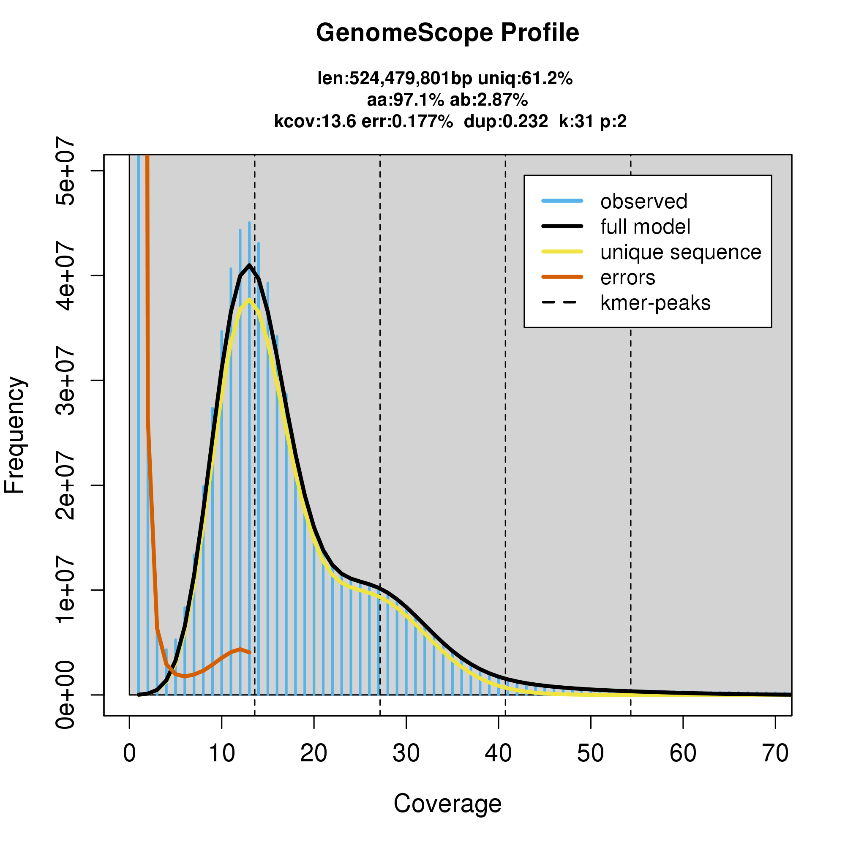

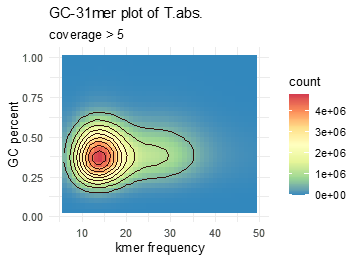


**A**

**B**

Figure S2: Quality assessment of the genome assembly using Merqury. K-mer multiplicity plots by Merqury of the hifiasm primary assembly (A) before and (B) after purging retained haplotigs. (C) Table of Lepidopteran BUSCO scores of the purged and unpurged hifiasm primary assembly, compared to the previously published T. absoluta assembly (Tabuloc et al. 2019).


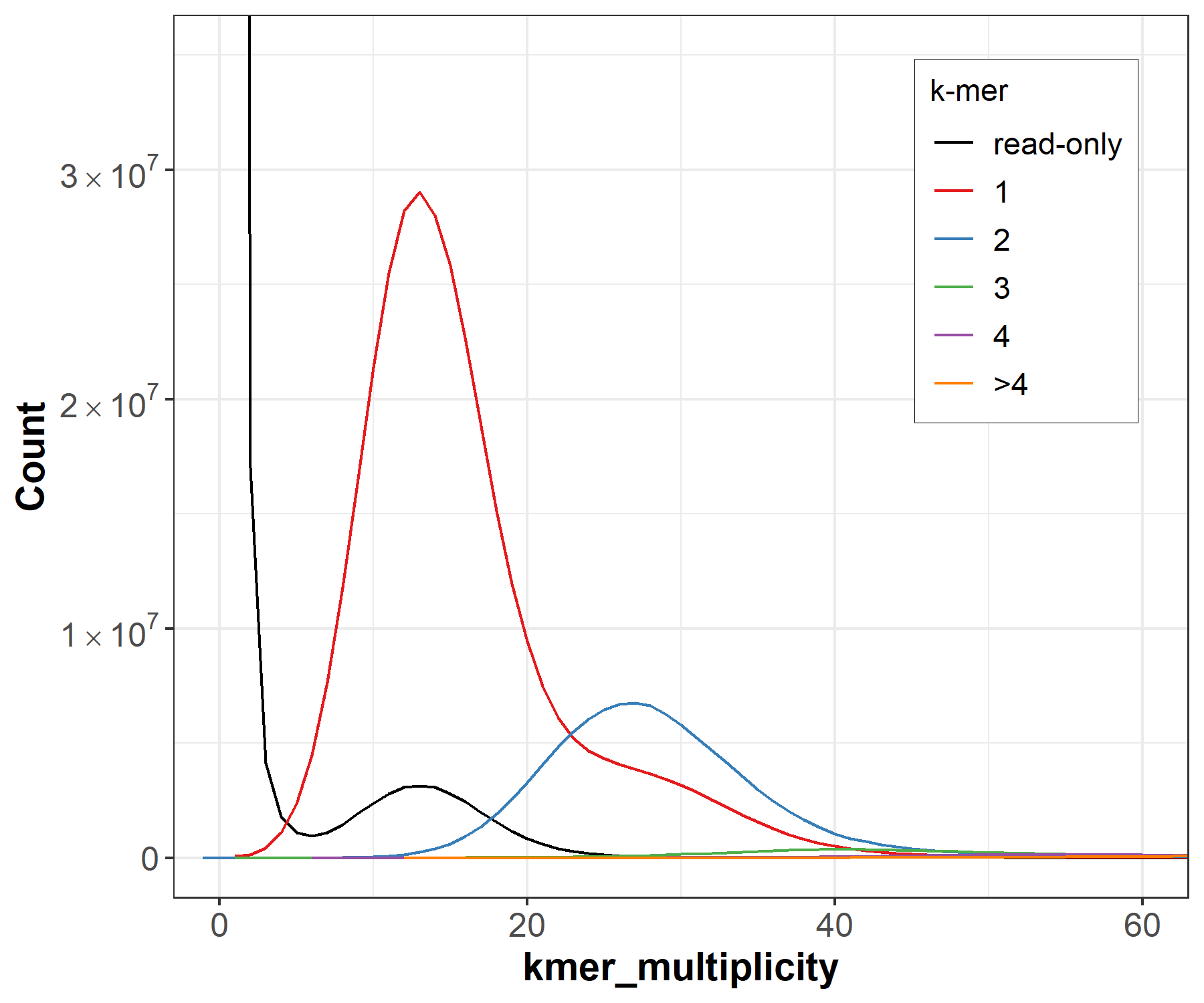

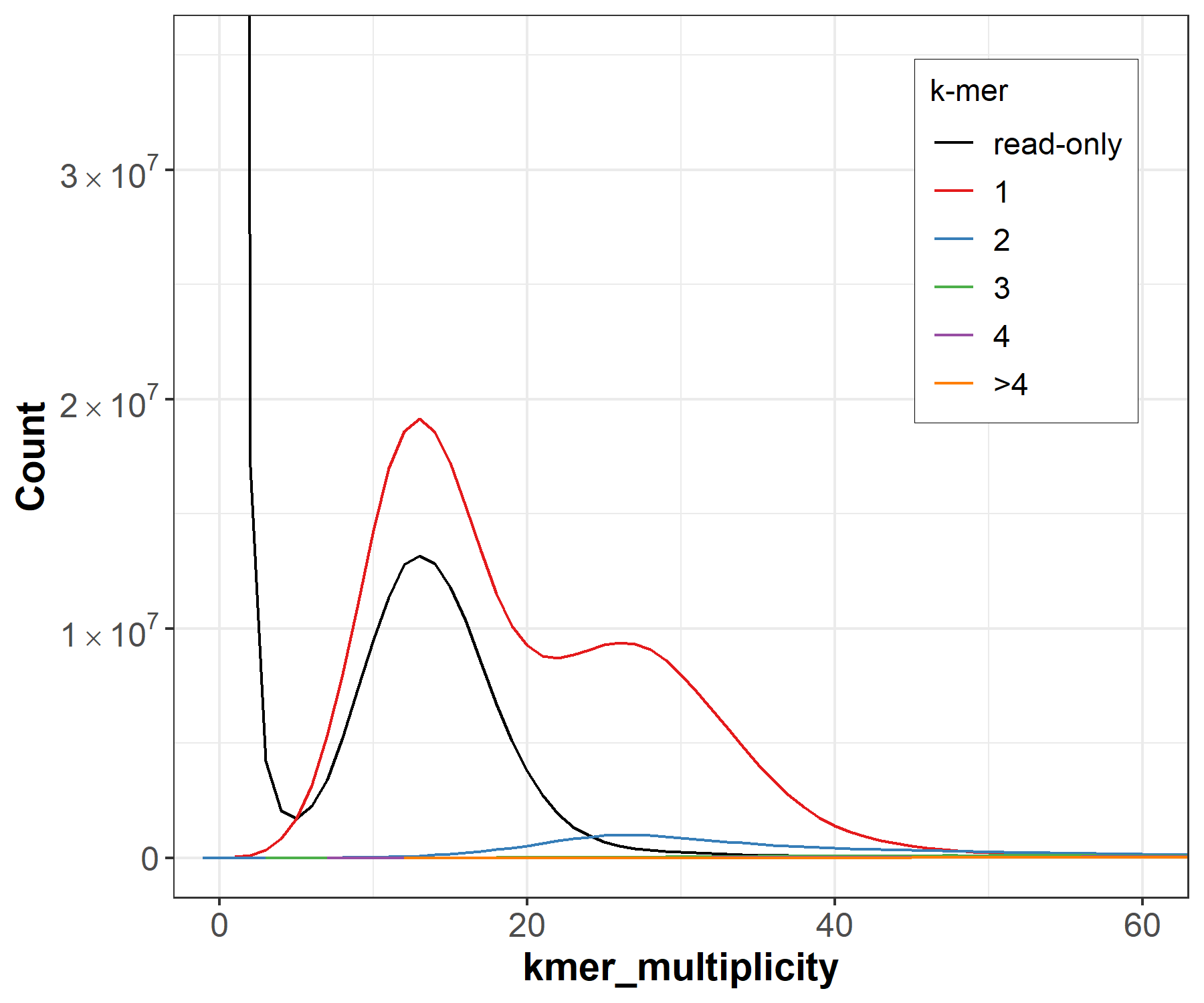


**A**

**B**


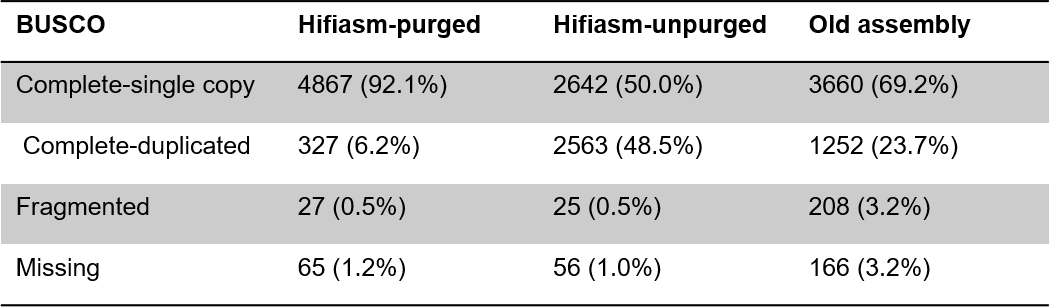


**C**

Figure S3: Decontamination results of the primary assembly. (A) Blobplot of primary contigs showing best BLAST matches vs GC % and mean read depth. The three “Lepidopteran” contigs at the 30% GC position were contigs ptg000311l, pg000280l, and ptg000281l, which appear to be microsporidian contamination that has been mislabeled as Lepidopteran. B) Repeat content of each contig in the primary assembly. Note contigs ptg000213, ptg000280, and ptg000281 have abnormally low repeat content.

**A**


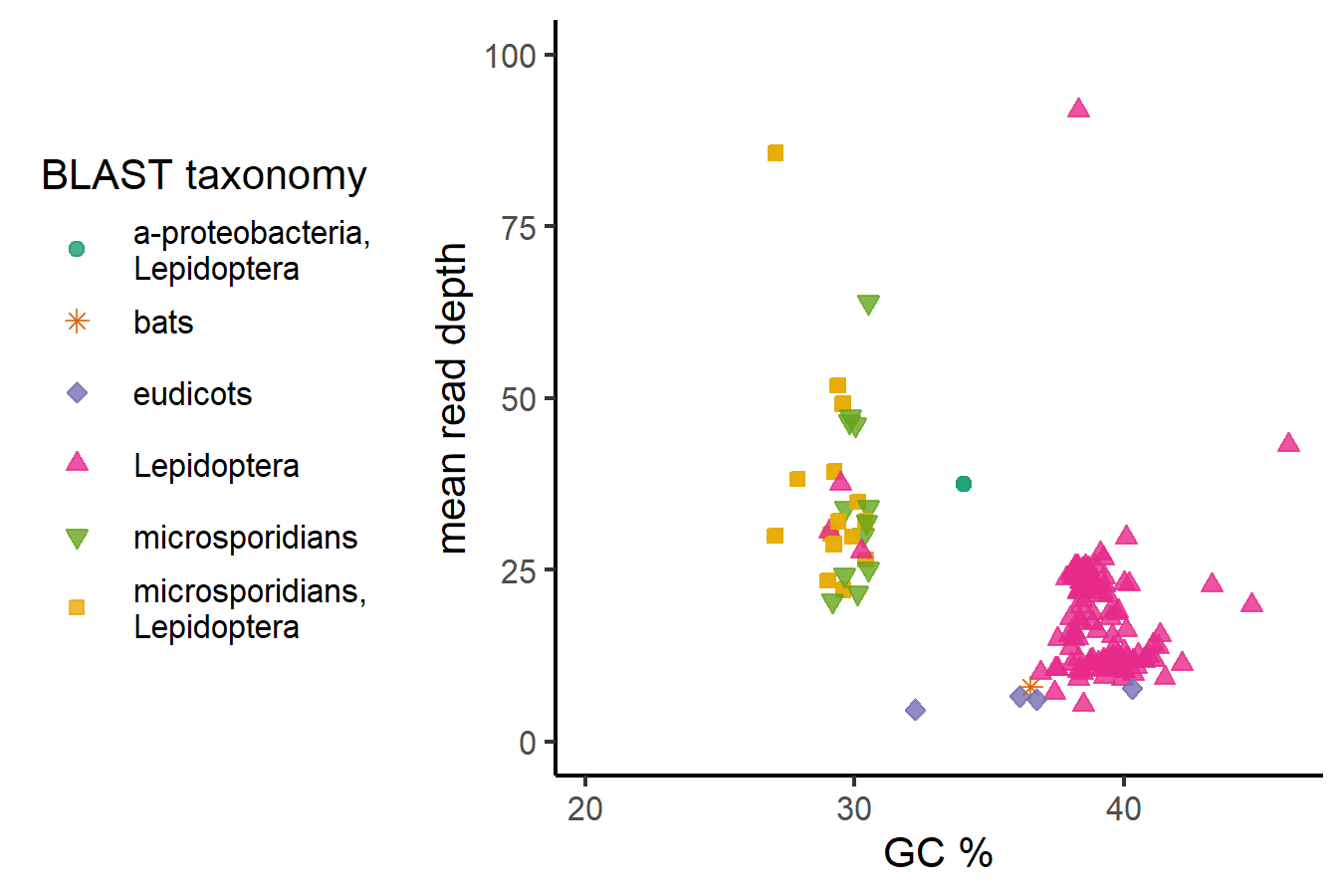

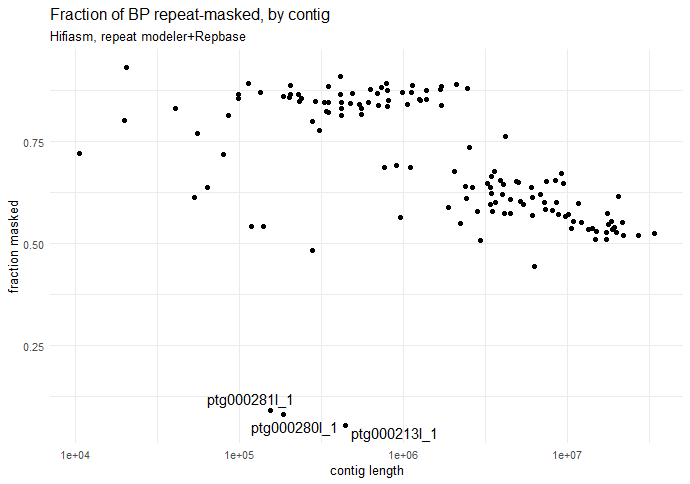


**B**

Figure S4: (A) Mapping rates and (B) median read coverage for Illumina reads from population sampling. Median read coverage was calculated at GC=39%, as this is the average GC content of the genome.


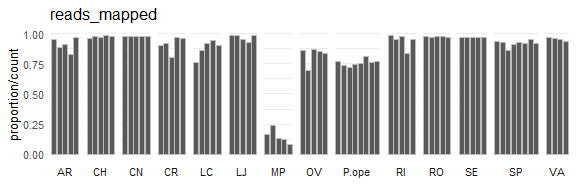

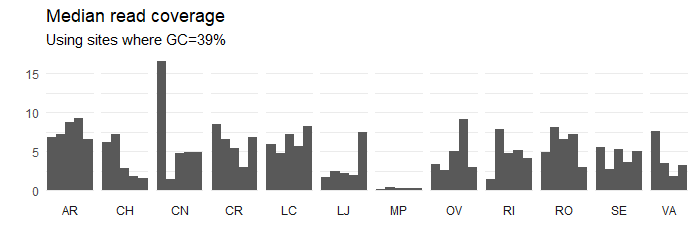


**A**

**B**

Figure S5: Summary statistics for the four populations detected using PCAngsd and NGSadmix. (A) Pairwise nucleotide diversity and (B) Tajima’s D for each cluster, averaged across the genome in 20kb windows. N represents the number of windows included. Pairwise t-tests with a Holm’s correction method were used to compare means. **** indicates p-value < 0.0001. (C) Unweighted Fst and (D) weighted Fst calculated by Angsd. Weighted Fst is typically considered more accurate as it is less biased when using many rare, population-specific SNPSs (as is the case when genotyping by whole-genome sequencing) (Bhatia et al. 2013).


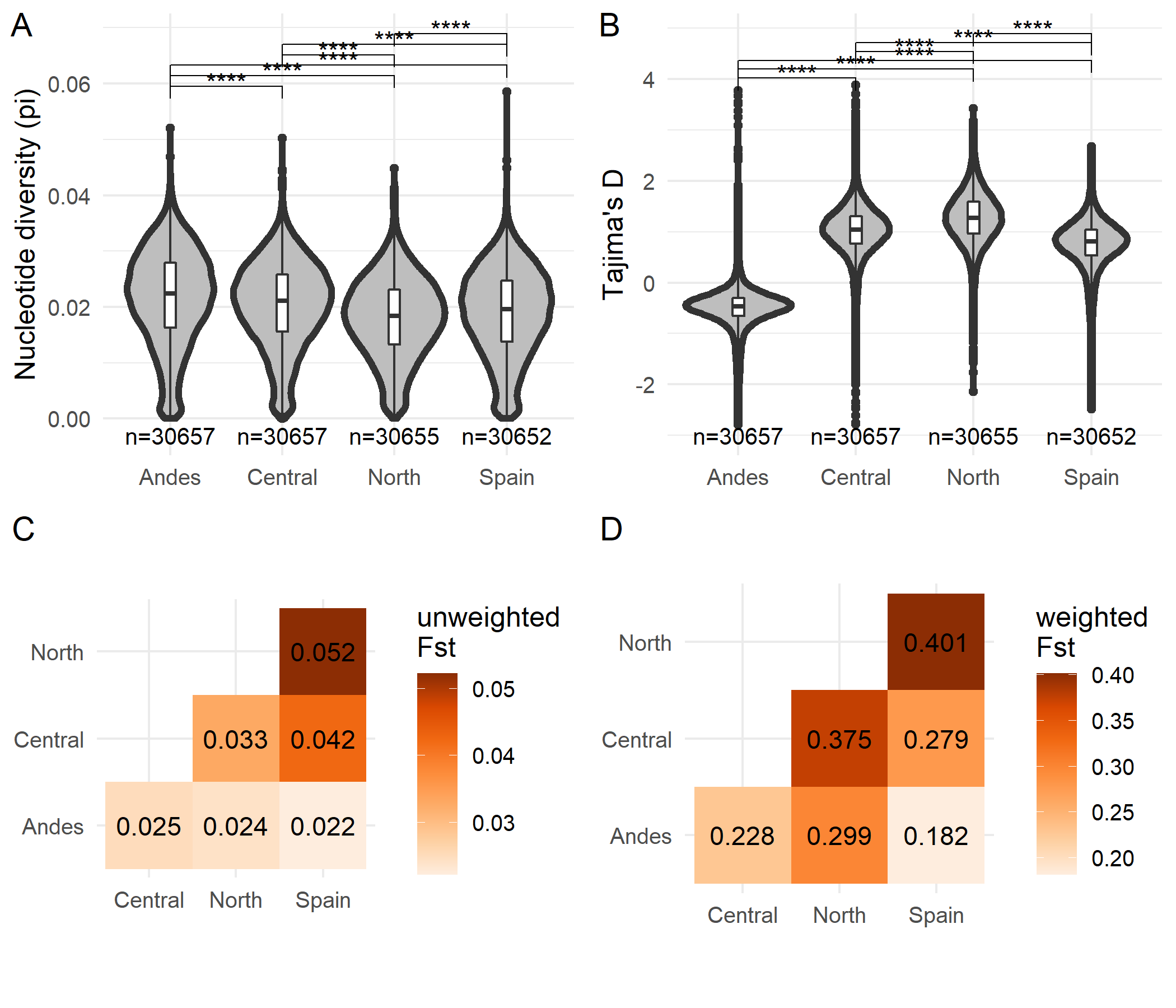


Figure S6: Three population models used to estimate parameter values. (A) M1: Model with two population splits and constant population size. (B) M2: Model with exponential growth. (C) M3: Model with population resizing events instead of exponential growth.


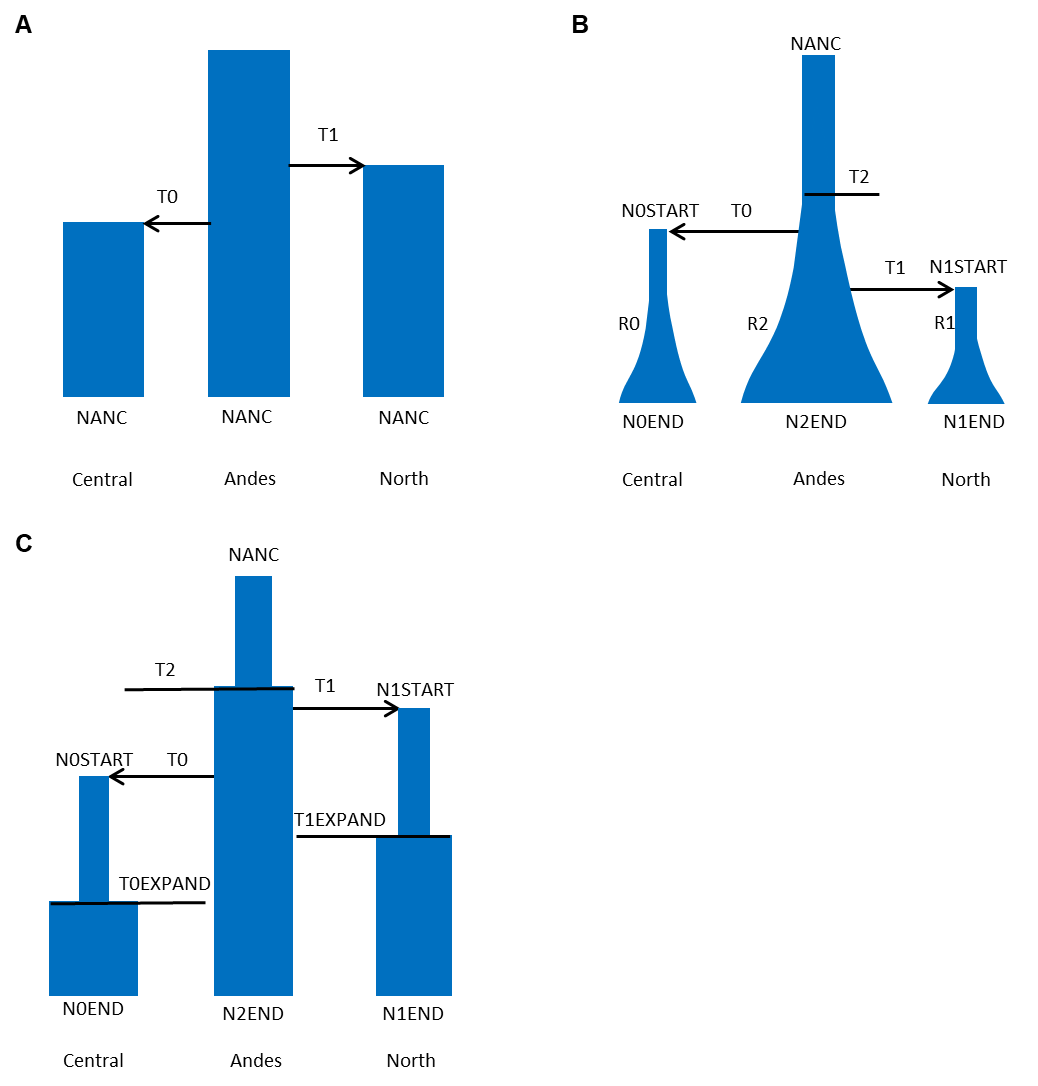


Figure S7: Linkage disequilibrium decay rates, minimums, and maximums over a 10kb interval calculated from genotype likelihoods or from 100 independent simulations under three different population history models. Values estimated from the data are shown by the red points with 95% confidence intervals. Pairwise t-tests with a Holm’s correction method were used to compare means. **** indicates p-value < 0.0001.


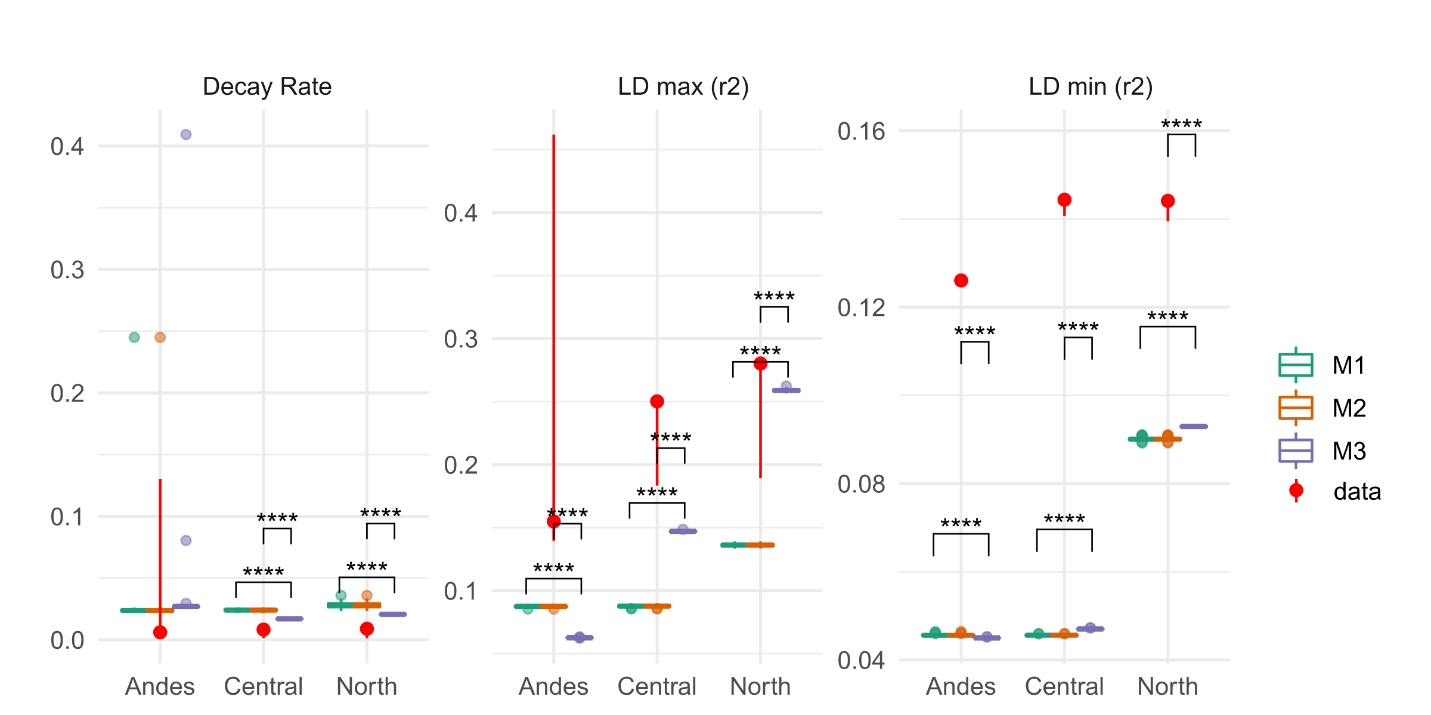


Figure S8: PBS and nucleotide diversity at three other PBS hotspots, with values averaged over 5kb intervals in 500bp steps.


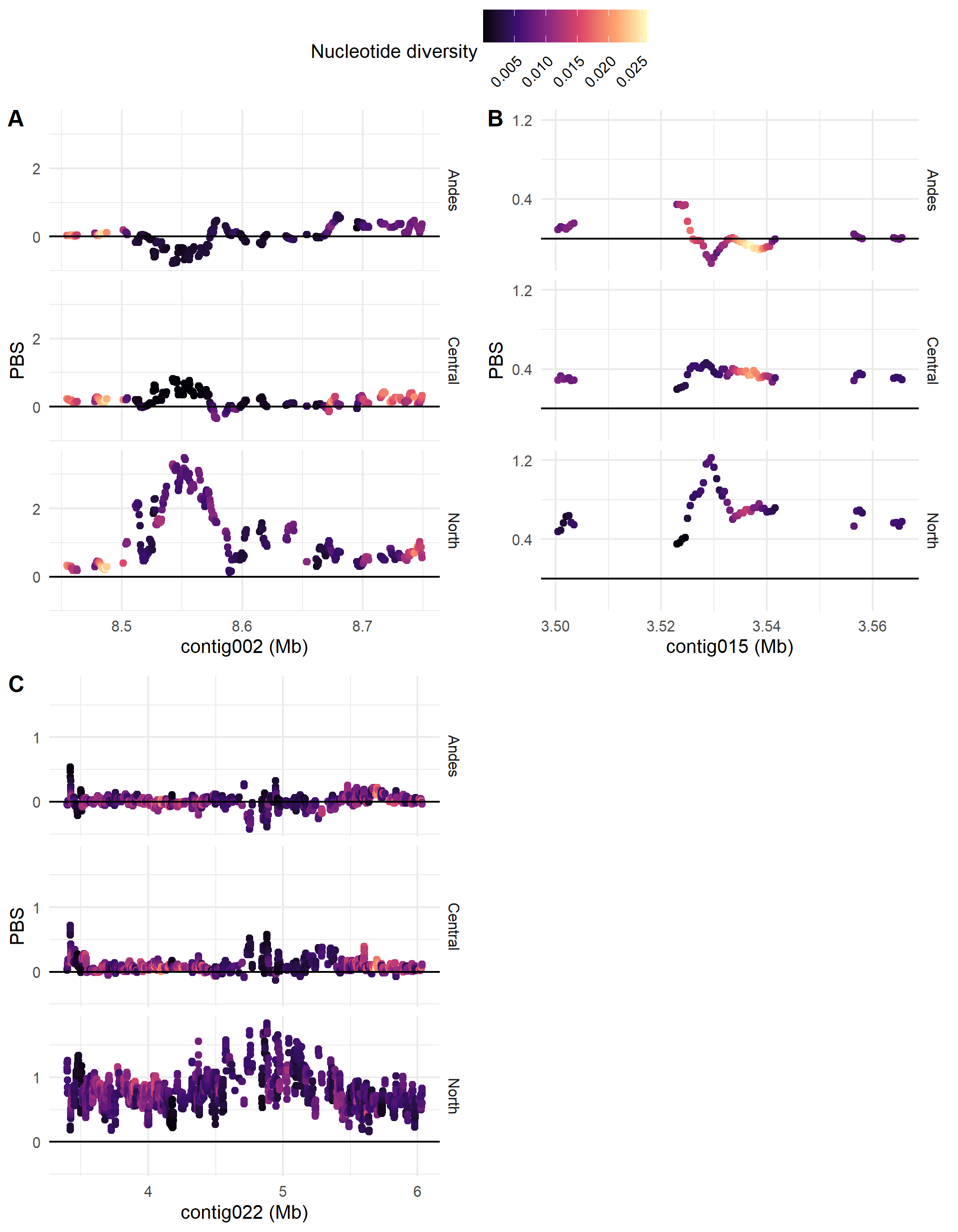

Supplement: evad060_Supplementary_Data [file evad060_supplementary_data.zip › Tuta absoluta popgen manuscript GBE SUPP Figures.docx]
